# The critical role of glutamine and fatty acids in the metabolic reprogramming of *anoikis*-resistant melanoma cells

**DOI:** 10.3389/fphar.2024.1422281

**Published:** 2024-08-08

**Authors:** S. Peppicelli, T. Kersikla, G. Menegazzi, E. Andreucci, J. Ruzzolini, C. Nediani, F. Bianchini, L. Calorini

**Affiliations:** Department of Experimental and Clinical Biomedical Sciences, University of Florence, Florence, Italy

**Keywords:** *anoikis* resistance, cell metabolism, circulating tumor cells, melanoma, therapy

## Abstract

**Introduction:** Circulating tumor cells (CTCs) represent the sub-population of cells shed into the vasculature and able to survive in the bloodstream, adhere to target vascular endothelial cells, and re-growth into the distant organ. CTCs have been found in the blood of most solid tumor-bearing patients and are used as a diagnostic marker. Although a complex genotypic and phenotypic signature characterizes CTCs, the ability to survive in suspension constitutes the most critical property, known as resistance to *anoikis*, e.g., the ability to resist apoptosis resulting from a loss of substrate adhesion. Here, we selected melanoma cells resistant to *anoikis*, and we studied their metabolic reprogramming, with the aim of identifying new metabolic targets of CTCs.

**Methods:** Subpopulations of melanoma cells expressing a high *anoikis*-resistant phenotype were selected by three consecutive rocking exposures in suspension and studied for their phenotypic and metabolic characteristics. Moreover, we tested the efficacy of different metabolic inhibitors targeting glycolysis (2DG), LDHA (LDHA-in-3), the mitochondrial electron transport chain complex I (rotenone), glutaminase (BPTES), fatty acid transporter (SSO), fatty acid synthase (denifanstat), CPT1 (etomoxir), to inhibit cell survival and colony formation ability after 24 h of rocking condition.

**Results:** *Anoikis-*resistant cells displayed higher ability to grow in suspension on agarose-covered dishes respect to control cells, and higher cell viability and colony formation capability after a further step in rocking condition. They showed also an epithelial-to-mesenchymal transition associated with high invasiveness and a stemness-like phenotype. *Anoikis*-resistant melanoma cells in suspension showed a metabolic reprogramming from a characteristic glycolytic metabolism toward a more oxidative metabolism based on the use of glutamine and fatty acids, while re-adhesion on the dishes reversed the metabolism to glycolysis. The treatment with metabolic inhibitors highlighted the effectiveness of rotenone, BPTES, SSO, and etomoxir in reducing the viability and the colony formation ability of cells capable of surviving in suspension, confirming the dependence of their metabolism on oxidative phosphorylation, using glutamine and fatty acids as the most important fuels.

**Discussion:** This finding opens up new therapeutic strategies based on metabolic inhibitors of glutaminase and fatty acid oxidation for the treatment of CTCs and melanoma metastases.

## 1 Introduction

Metastasis, the major cause of cancer mortality, represents the end of a multi-step process that includes detachment of cancer cells from the primary tumor, survival in the body’s vascular system, arrest, and proliferation in distant organs. Once tumor cells reach the circulatory system (circulating tumor cells, CTCs) they are exposed to several stresses and, in order to survive and metastasize, they need to overcome the loss of adhesion, which typically induces a type of programmed cell death known as *anoikis*. *Anoikis*, from the Greek word “homelessness,” is an important mechanism for maintaining tissue homeostasis, preventing adherent-independent cell growth or attachment to an inappropriate matrix (Taddei et al., 2012). It follows that tumor cells, which need to survive after detaching themselves from the primary tumor and gain lymphatic or blood circulation, must necessarily acquire the ability to resist *anoikis.* Only tumor cells that acquire this anchorage-independent survival mechanism, can complete the metastatic cascade; thus, resistance to *anoikis* can be considered a requirement for metastasizing CTCs (Paoli et al., 2013; Khan et al., 2022). *Anoikis* resistance is under the control of many different factors, such as cell adhesion molecules, growth factors, oxidative stress, signaling pathways, and biochemical and molecular alterations within the cell milieu (Adeshakin et al., 2021). Several studies have highlighted the link between *anoikis* resistance and the epithelial-to-mesenchymal transition (EMT) (Cao et al., 2016), a process in which epithelial cells remodel the cytoskeleton, detach from neighboring cells, and become mobile, invasive, and resistant to apoptotic stimuli (Brabletz et al., 2018). EMT plays a critical role in tumor metastasis and is the most investigated phenotypic characteristic of CTCs (Lawrence et al., 2023). In recent years, accumulating evidence supports the correlation between the metabolic adaptation that cancer cells undergo after ECM detachment and their ECM-independent survival (Mason et al., 2017; Hawk and Schafer, 2018; Endo et al., 2020), thus making the metabolic regulation of *anoikis* a topic of great interest in cancer research.

Metabolic reprogramming is a hallmark of cancer essential for cancer cells to adapt to the dynamic nutritional conditions of the tumor microenvironment during cancer progression (Faubert et al., 2020). Since the first disclosure in the 1920s by the physiologist Otto Warburg, who observed that cancer cells prefer glycolysis rather than mitochondrial oxidative phosphorylation (OXPHOS) even under sufficient oxygen supply (Warburg effect), several recent studies have demonstrated that malignant cells exhibit metabolic plasticity switching between glycolysis and OXPHOS, and the opposite, depending on microenvironmental conditions (Jose et al., 2011; Shuvalov et al., 2021). As this metabolic conversion depends on the bioenergetic and biosynthetic demands and on the redox homeostasis required to sustain specific behavior of tumor cells (such as proliferation, invasiveness, and survival) (DeBerardinis and Chandel, 2016), it is expected that viable CTCs could be characterized by a metabolic phenotype different from that of proliferating tumor cells of the primary tumor mass.

Although some recent studies have highlighted the importance of metabolic deregulation to allow CTC to bypass *anoikis*, it is still unclear the metabolic adaptation on which surviving detached tumor cells depend.

Our study aims to disclose the metabolic reprogramming of *anoikis-*resistant melanoma cells to identify novel therapeutic approaches to eradicate CTCs and prevent metastasis.

Here, we demonstrate that *anoikis*-resistant melanoma subpopulations, obtained after three consecutive rocking exposures in suspension, show a mesenchymal phenotype with high invasive ability, associated with a metabolic reprogramming from a characteristic glycolytic metabolism toward a more oxidative metabolism based on the use of glutamine and fatty acids. Our results also showed that the inhibition of glutaminase, fatty acid transporters, or beta-oxidation during the rocking condition in suspension was effective in reducing the viability and colony-efficiency of our *anoikis*-resistant melanoma cells, opening up new possibilities for the treatment of CTCs and to counteract melanoma dissemination.

## 2 Materials and methods

### 2.1 Cell lines and culture conditions

In this study, we used the melanoma cell lines A375M6, isolated in our laboratory as described in our previous work (Ruzzolini et al., 2017), SKMel2 obtained from ATCC, SKMel28 kindly provided by Dr Laura Poliseno (CNR, Pisa, Italy).

In some experiments, we also used the adenocarcinomic human alveolar basal epithelial A549 and the pancreatic cancer cells PANC1 (kindly provided by Dr. Anna Laurenzana, University of Florence, Italy). Melanoma cells were cultivated in Dulbecco’s Modified Eagle Medium high glucose (DMEM 4500, EuroClone, MI, Italy) supplemented with 10% foetal bovine serum (FBS, Boehringer Mannheim, Germany), at 37^°^C in a humidified atmosphere containing 90% air and 5% CO_2_. A549 and PANC1 cells were cultivated in RPMI-1640 medium (EuroClone) supplemented with 10% FBS, and 2 mM glutamine (EuroClone), at 37^°^C in a humidified atmosphere containing 90% air and 10% CO_2_. Cells were harvested from subconfluent cultures by incubation with a trypsin-EDTA solution (EuroClone) and propagated every 3 days. The viability of the cells was determined by Trypan blue exclusion test. Cultures were periodically monitored for mycoplasma contamination.

To select melanoma cells with a well-consolidated *anoikis*-resistant phenotype, A375M6, SkMel28, and SkMel2 melanoma cells were exposed several times to a loss of adherence condition, as shown in Supplementary Figure 1. In particular, 5 × 10^4^ melanoma cells suspended in a growth factor-free Dulbecco’s D-MEM Nutrient mix F12 medium (DME/F12-HEPES EuroClone) were placed in sterile non-adherent 50 mL tubes, that were shacked on a Mini rocker platform shaker (20°angle) (Biosan, Riga, Latvia) for 24 h, at 37°C (Peppicelli et al., 2019). Cells were recovered and placed on an adherent plastic dish to grow until reaching an adequate number to be used to proceed with further rocking exposure. At the end of the third rocking exposure (p2-Suspension, p2-S), recovered cells were grown in an adherent plastic dish to obtain the p3-Adhesion population.

### 2.2 *Anoikis* assay

To simulate anchorage-independent growth conditions, we cultured melanoma cells in tubes in rocking conditions (Peppicelli et al., 2019). 5 × 10^4^ cells were left rocking in tubes on the Mini Rocker Shaker (Biosan, Riga, Latvia), at room temperature in Dulbecco’s D-MEM Nutrient mix F12 (DME/F12-HEPES EuroClone) for 24 h. In some experiments, during the rocking period, cells were treated with 2-Deoxy-D-glucose (2DG), a glucose analog that inhibits glycolysis (D8375, Merk); LDHA-IN-3, a potent lactate dehydrogenase A inhibitor (HY-139319, MedchemExpress, Stockholm, Sweden); rotenone, a mitochondrial electron transport chain complex I inhibitor (557,368, Sigma-Aldrich, Milan, Italy); BPTES, a selective glutaminase inhibitor (HY-12683, MedchemExpress), Denifanstat, a Fatty Acid Synthase (FASN) inhibitor (HY-112829, MedchemExpress); Etomoxir, an irreversible inhibitor of carnitine palmitoyltransferase 1a (CPT-1a), inhibiting fatty acid oxidation (FAO) (HY-50202, MedchemExpress); Sulfo-N-succinimidyl Oleate, a long chain fatty acid that inhibits fatty acid transport into cells (SML2148, Sigma-Aldrich). After 24 h of rocking, cells were used for Western blot analysis, invasion assay, live and dead assay, or colony formation assay.

To test *anoikis* resistance melanoma cells were cultured in dishes coated with agarose. Culture dishes were coated with 1.5% agarose (Promega, San Luis Obispo, California). 5 × 10^4^ cells were plated in agarose-coated petri dishes in complete medium and after 3 or 7 days, the cells were photographed.

### 2.3 Annexin V/PI flow cytometer analysis

Apoptosis was measured by flow cytometry, using the APC-conjugated Annexin V staining. Cells incubated in rocking condition for 24 h were collected, washed with PBS, and resuspended in 100 μL of 1x Annexin-binding buffer (100 mM HEPES, 140 mM NaCl, 25 mM CaCl2, pH 7.4), with 3 µL of Annexin V APC-conjugated (ImmunoTools, Friesoythe, Germany) and 1 µL of 100 μg/mL propidium iodide (PI, P4864, Sigma-Aldrich) working solution. After 15 min of incubation at 4°C in the dark condition 400 μL of 1X Annexin Binding Buffer was added to each sample and cells were analyzed by flow cytometry (BD-FACS Canto) to find out the viability (annexin V-and PI ^-^, Q3), early apoptosis (annexin V ^+^ and PI ^-^, Q4), or late apoptosis (annexin V ^+^ and PI ^+^, Q2). A minimum of 5,000 events were collected.

### 2.4 Colony formation assay

After 24 h in rocking conditions, 5 × 10^3^ cells were transferred to a culture dish in fresh medium and incubated for 10 days at 37°C. The colonies were washed with PBS, fixed in cold methanol, and stained using a Diff Quik kit (BD Biosciences, distributed by DBA, Milan, Italy). The stained colonies were photographed with a digital camera and the number of colonies in each well was counted.

### 2.5 Western blotting analysis

Cells were washed with ice-cold PBS containing 1 mM Na4VO3 and lysed in 100 *μ*L of cell RIPA lysis buffer (Merck Millipore, Vimodrone, MI, Italy) containing PMSF (Sigma-Aldrich), sodium orthovanadate (Sigma-Aldrich), and protease inhibitor cocktail (Merck Millipore).

The protein concentration was measured using Bradford reagent (Merck Millipore), and aliquots of supernatants containing equal amounts of protein were separated in Laemmli buffer on Bolt Bis-Tris Plus gels 4%–12% precast polyacrylamide gels (Life Technologies, Monza, Italy). Fractionated proteins were transferred from the gel to a polyvinylidene difluoride (PVDF) membrane using the iBlot 2 System (Thermo Fischer Scientific, Milan, Italy). Blots were blocked for 5 min, at room temperature, with the EveryBlot Blocking Buffer (BioRad). Subsequently, the membrane was probed at 4^°^C overnight with primary antibodies diluted in a solution of 1:1 Immobilon^®^ Block – FL (Merck Millipore)/T-PBS buffer. The primary antibodies were as follows: rabbit anti-GLUT-3, rabbit anti-HK2, rabbit anti-PKM2, rabbit anti-FASN, rabbit anti-ACSL, mouse anti-vinculin (1:1,000 Cell Signalling Technology, Danvers, MA, United States), mouse anti-N-Cadherin (1:1,000 DAKO Agilent, Milan, Italy), rabbit anti-IKB alpha (1:1,000 Abcam, Cambridge, United Kingdom), rabbit anti-MCT1, mouse anti-LDHB (Santa Cruz Biotechnology), mouse anti-tubulin antibody (1:2,000, GeneTex, Alton Pkwy Irvine, CA, United States). The membrane was washed in T-PBS buffer, incubated for 1 h with goat anti-rabbit IgG Alexa Fluor 750 antibody or with goat anti-mouse IgG Alexa Fluor 680 antibody (Invitrogen, Monza, Italy), and then visualized by an Odyssey Infrared Imaging System (LI-COR Bioscience). Mouse anti-tubulin or mouse anti-vinculin antibodies were used to assess an equal amount of protein loaded in each lane.

### 2.6 Quantitative real-time PCR

Total RNA was extracted from cells using Tri Reagent (Cat. No. T9424, Sigma-Aldrich), agarose gel checked for integrity, and 1 μg of total RNA was reverse transcribed with iScript cDNA Synthesis Kit (Cat. No. 1708891, BioRad) according to the manufacturer’s instructions. Quantitative real-time PCR (qPCR) was performed using the Sso Advanced Universal SYBR Green Supermix (Cat. No. 1725274, BioRad). The qPCR analysis was carried out in triplicate with a CFX96 Real-Time PCR System (BioRad) with the default PCR setting: 40 cycles of 95°for 10 s and 60°C for 30 s. The fold change was determined by the comparative Ct method using 18S and β-actin as reference genes. Primer sequences are reported in Table 1.

**TABLE 1:** List of primers used for quantitative real-time PCR.

Gene	Primer FW	Primer REV
18s	5′-cgc​cgc​tag​agg​tga​aat​tct-3′	5′-cgaacctccga ctttcgttct-3′
ACC1	5′-ttc​act​cca​cct​tgt​cag​cgg​a-3′	5′-gtc​aga​gaa​gca​gcc​cat​cac​t-3′
ACC2	5′-gac​gag​ctg​atc​tcc​atc​ctc​a-3′	5′-atg​gac​tcc​acc​tgg​tta​tgc​c-3′
ALDH1A1	5′-ggt​ggc​tgg​caa​gat​cgt-3′	5′-cca​agg​cgg​gca​aag​ag-3′
ASCT2	5′- ggtggctggcaagatcgt -3′	5′- ccaaggcgggcaaagag -3′
β-actin	5′-tcg​agc​cat​aaa​agg​caa​ct-3′	5′-ctt​cct​caa​tct​cgc​tct​cg-3′
CytC	5′-ttg​cac​tta​cac​cgg​tac​tta​agc-3′	5′-acg​tcc​cca​ctc​tct​aag​tcc​aa-3′
COX5B	5′-tgc​gct​cca​tgg​cat​ct-3′	5′-ccc​agt​cgc​ctg​ctc​ttc-3′
CPT1	5′-gat​cct​gga​caa​tac​ctc​gga​g-3′	5′-ctc​cac​agc​atc​aag​aga​ctg​c-3′
CPT2	5′-gca​gat​gat​ggt​tga​gtg​ctc​c-3′	5′-aga​tgc​cgc​aga​gca​aac​aag​tg-3′
FATP1	5′-tga​cag​tcg​tcc​tcc​gca​aga​a-3′	5′-ctt​cag​cag​gta​gcg​gca​gat​c-3′
FATP5	5′-gga​agt​cta​cgg​ctc​cac​aga​a-3′	5′-gtc​gaa​ctg​cac​cag​ctc​aaa​g-3′
GLUT1	5′-cgggccaagagtgtg ctaaa-3′	5′-tga​cga​tac​cgg​agc​caa​tg-3′
GLUT3	5′-cga​act​tcc​tag​tcg​gat​tg-3′	5′-agg​agg​cac​gac​tta​gac​at-3′
GLS1	5′-tgc​tac​ctg​tct​cca​tgg​ctt-3′	5′- ctt​aga​tgg​cac​ctc​ctt​tgg -3′
GLS2	5′-tgc​cta​tag​tgg​cga​tgt​ctc​a-3′	5′-gtt​cca​tat​cca​ggc​tga​caa-3′
HK2	5′-caa​agt​gac​agt​ggg​tgt​gg-3′	5′-gcc​agg​tcc​ttc​act​gtc​tc-3′
KFL4	5′-gca​gcc​acc​tgg​cga​gtc​tg-3′	5′-ccg​cca​gcg​gtt​att​cgg​gg-3′
LDHA	5′-agc​ccg​att​ccg​tta​cct-3′	5′-cac​cag​caa​cat​tca​ttc​ca-3′
LDHB	5′-cta​gat​ttc​gct​acc​tta​t-3′	5′-tca​ttg​tca​gtt​ccc​att-3′
MCT1	5′-cca​aga​cct​cgt​gtt​gag​acc-3′	5′-aat​aca​gct​cag​gtc​tcc​ttg​g-3′
N-Caderina	5′-cac​tgc​tca​gga​ccc​aga​t-3′	5′-taa​gcc​gag​tga​tgg​tcc-3′
OCT3/4	5′-ttt​tgg​tac​ccc​agg​cta​tg-3′	5′-gca​ggc​acc​tca​gtt​tga​at-3′
PKM2	5′-cag​agg​ctg​cca​tct​acc​ac-3′	5′-cca​gac​ttg​gtg​agg​acg​at-3′
Snail1	5′-ccc​agt​gcc​tcg​acc​act​at-3′	5′-cca​gat​gag​cat​tgg​cag-3′
SOX2	5′-gag​ctt​tgc​agg​aag​ttt​gc-3′	5′-gca​aga​agc​ctc​tcc​ttg​aa-3′
Vimentina	5′-tgt​cca​aat​cga​tgt​gga​tgt​ttc-3′	5′-ttg​tac​cat​tct​tct​gcc​tcc​tg-3′
Zeb1	5′-cgc​cgc​tag​agg​tga​aat​tct-3′	5′-cgaacctccga ctttcgttct-3′

### 2.7 Invasion assay

Invasiveness of melanoma cells was determined *in vitro* on Matrigel (BD Biosciences) precoated polycarbonate filters, with 8 *μ*m pore size, 6.5 mm diameter, 12.5 *μ*g Matrigel/filter, mounted in Boyden’s chambers. 1.5 × 10^5^ cells (200 *μ*L) were seeded in their growth medium in the upper compartment and incubated for 6 h at 37°C in 10% CO_2_ in air. In the lower chamber, a complete medium was added as a chemoattractant. After incubation, the inserts were removed and the non-invading cells on the upper surface were wiped off mechanically with a cotton swab and the membranes were fixed overnight in ice-cold methanol. Cells on the lower side of the membranes were then stained using the Diff-Quick kit (BD Biosciences) and photographs of randomly chosen fields were taken.

### 2.8 Live and dead assay

The LIVE/DEAD™ Fixable Violet Dead Cell Stain Kit (L34964, Invitrogen) was used to determine the viability of cells incubated for 24 h in rocking conditions or treated with inhibitors. Briefly, 0.5 μL of diluted stain was added to 0.5 mL PBS in a flow cytometer tube containing 1 × 10^5^ cells. Cells and stains were mixed and incubated for 30 min at room temperature. Cells were washed and analyzed on a flow cytometer.

### 2.9 Cell cycle analysis

Cell cycle distribution was analyzed by the DNA content using PI staining method. Cells, incubated for 24 h in rocking condition, were washed 2 times in PBS by centrifugation and stained with a mixture of 50 μg/mL propidium iodide (PI, P4864, Sigma-Aldrich), 20 μg/mL RNase A, 1 mg/mL trisodium citrate and 0.3% (v/v) Triton X-100 in the dark at room temperature for 30 min. The stained cells were analyzed by flow cytometry (BD-FACS Canto) using red propidium-DNA fluorescence.

### 2.10 Extracellular lactate measurement

For extracellular L-lactate determination, the supernatants of cells grown in adhesion or suspension were collected and centrifuged at 10,000 rpm for 5 min to remove insoluble particles. All sample preparation steps were performed at 4°C and samples were stored at −80°C until analysis. L-lactate concentrations were measured using the Lactate assay kit (colorimetric) (MET-5012, Cell Biolabs, Inc., San Diego, CA, United States) following the manufacturer’s instructions. The plate has been read with a spectrophotometric microplate reader at 540 nm.

### 2.11 Seahorse metabolic flux analyses

Seahorse analysis has been performed as previously described (Ruzzolini et al., 2020) using the Seahorse XF96 Extracellular Flux Analyzer (Seahorse Bioscience, Billerica, MA, United States). 3 × 10^4^ cells were seeded in XF96 Seahorse^®^ microplates precoated with poly-D-lysine (ThermoFisher Scientific, Waltham, MA, United States) in XF Assay Medium (Agilent Technologies, Santa Clara, CA, United States) supplemented with 2 mM glutamine. Mitochondrial stress tests (kit from Agilent Technologies), were performed to determine real-time Oxygen Consumption Rate (OCR), while glycolytic rate assay tests (kit from Agilent Technologies) were performed to determine proton efflux rate in melanoma cells grown in adhesion or p3 cells after 24 h in rocking conditions. For the mitochondrial stress test inhibitors were used at the final concentrations of 1 μM oligomycin, 0.5 μM FCCP, and 0.5 μM rotenone/antimycin A. For the glycolytic rate test, Rotenon/antimycin A were used at 0.5 μM, 2DG at 50 mM. Substrate oxidation stress tests were performed to determine the specific substrates oxidated by cells grown in adherent conditions or by p3 cells after 24 h in rocking conditions. We considered the three primary substrates that fuel the mitochondria: long-chain fatty acids, glucose/pyruvate, and/or glutamine. The final concentrations of inhibitors were 3 μM BPTES, 4 μM etomoxir, and 2 μM UK5099. Normalization to protein content was performed after each experiment. The Seahorse XF Report Generator automatically calculated the parameters from Wave data that have been exported to Excel.

### 2.12 Statistic analysis

Densitometric data are expressed as means ± standard errors of the mean (SEM) depicted by vertical bars from representative experiment of at least three independent experiments.

Statistical analysis of the data was performed by Student’s t test or two-way ANOVA (when more than two samples were compared). Values of *p* ≤ 0.05 were considered statistically significant.

## 3 Results

### 3.1 Metabolic reprogramming of *anoikis*-resistant melanoma cells

**FIGURE 1:**
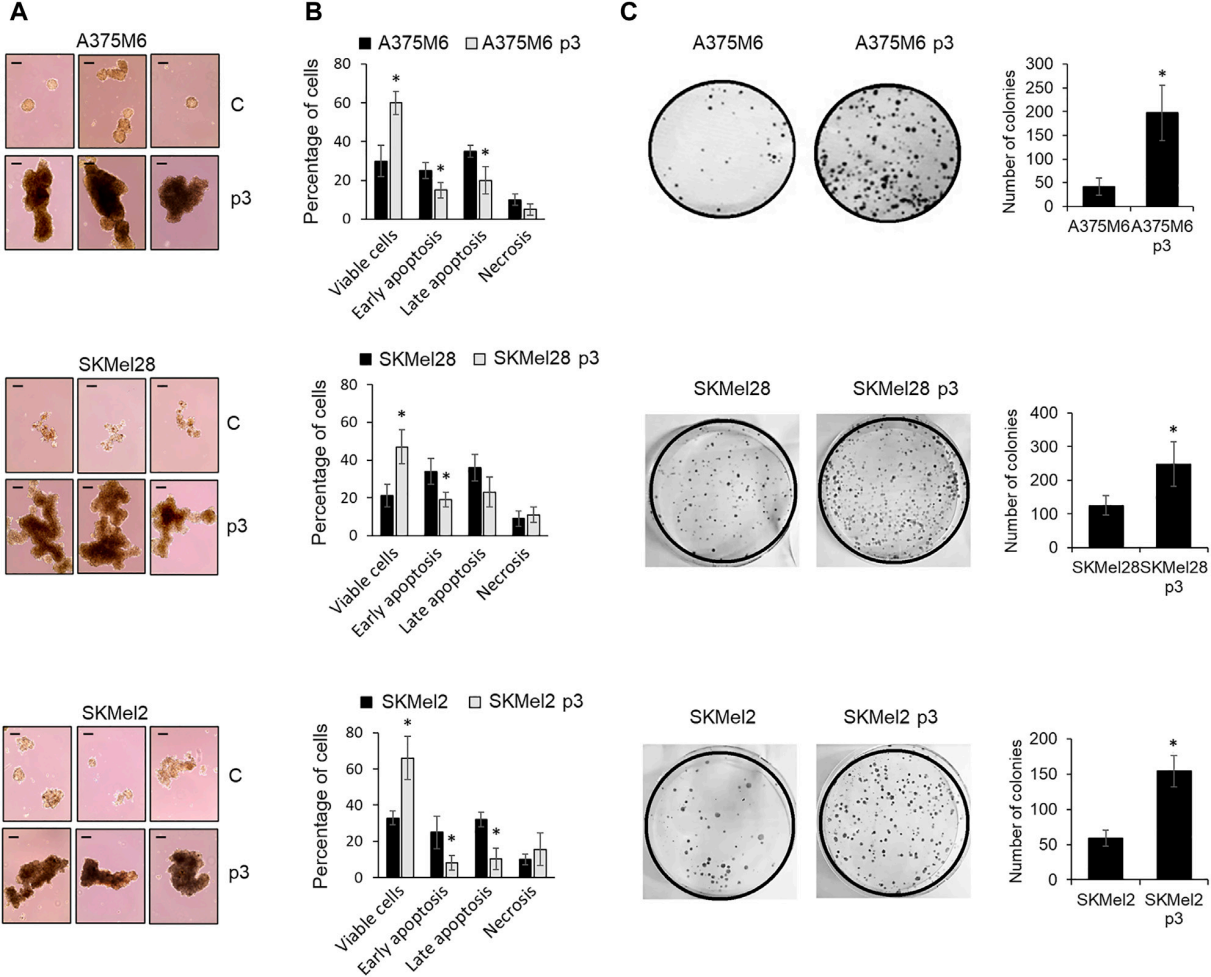
Evaluation of the *anoikis* resistance of p3 melanoma cells. **(A)** Three representative images of A375M6, SkMel28, SkMel2 melanoma cells (control vs*.* p3) allowed to growth for 7 days on agarose-coated dishes. Scale bar: 200 μm. **(B)** Flow cytometry analysis of cells apoptosis after staining with annexin V-APC/propidium iodide (PI), and **(C)** representative images of clonogenic efficiency (left) and their respective quantification expressed as the number of colonies (right) of melanoma cells (control vs*.* p3) after exposure to 24 h of rocking condition. *P < 0.05 compared with control cells.

To select melanoma cells with a well-consolidated *anoikis*-resistant phenotype, A375M6, SkMel28, and SkMel2 melanoma cells were exposed three times to a loss of adherence condition, as described in the “Material and Methods” section and shown in Supplementary Figure 1. At the end of the third rocking exposure (p2-Suspension, p2-S), recovered cells were grown in an adherent plastic dish (p3-Adhesion) and then tested for *anoikis* resistance, culturing cells on agarose-coated dishes. Agarose was used as a support to prevent cell adhesion, to mimic cell growth in suspension. Control cells or p3-Adh population distributed on agarose-coated dishes were grown for 7 days in a complete medium and the number of tumor cell aggregates was evaluated after 7 days. Figure 1A shows the higher ability of the p3 population respect to control cells to give rise to cell aggregates with a bigger diameter. To further prove the greater ability to resist *anoikis* acquired by the p3 population compared to the starting population, we also placed the cells again in a rocking condition for 24 h and then we evaluated cell viability, apoptosis induction, and colony-forming ability. The selected populations of melanoma cells (A375M6 p3, SKMel28 p3, SKMel2 p3) showed higher cell viability evaluated by live/dead cell staining (Supplementary Figure 2), a lower percentage of apoptotic cells (Figure 1B; representative Annexin V/PI plots are shown in Supplementary Figure S3) and higher colony efficiency respect to control cells (Figure 1C).

**FIGURE 2:**
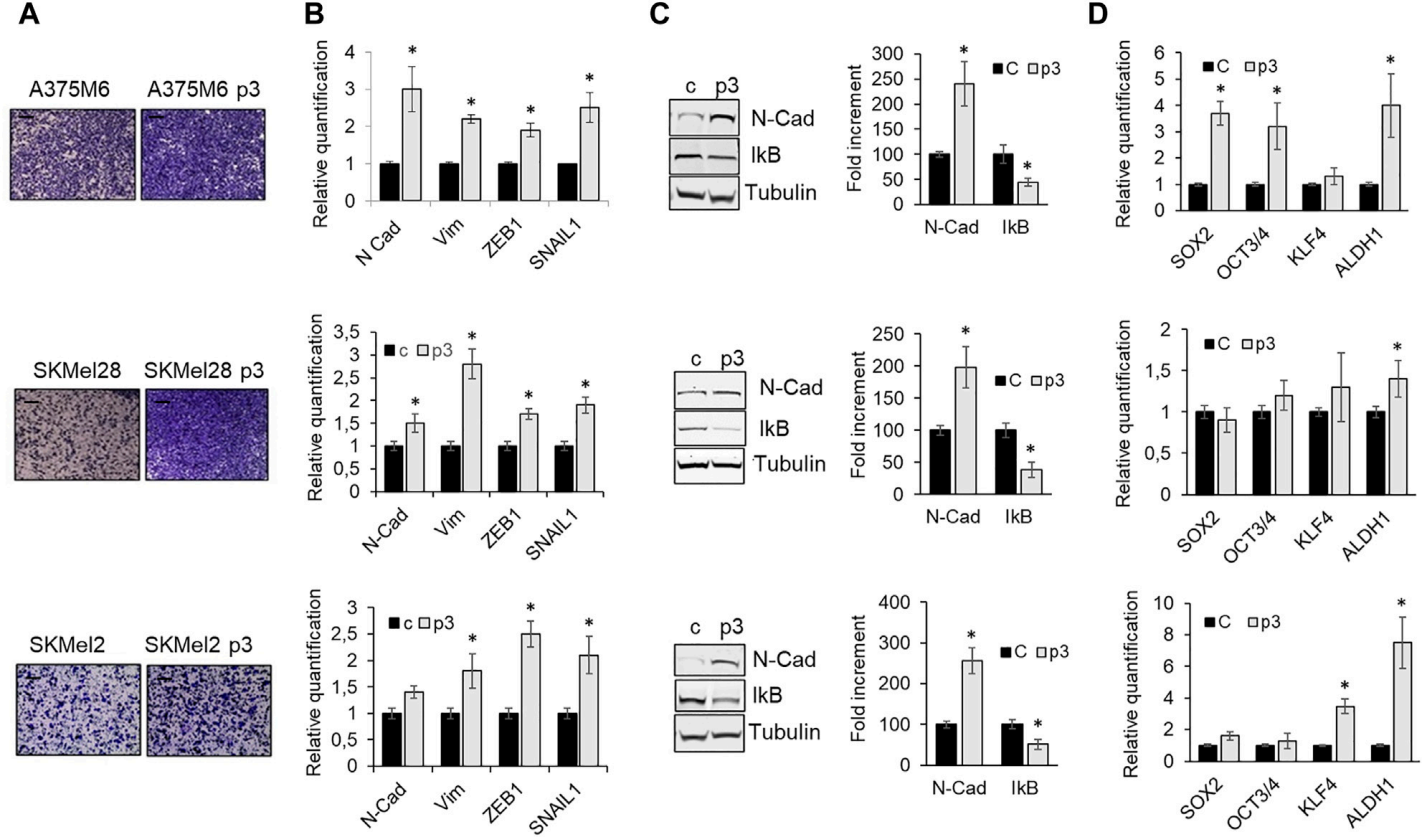
Phenotype of p3 melanoma cells. A375M6, SkMel28, SkMel2 melanoma cells were grown in standard conditions or selected by three consecutive rocking exposures in suspension (A375M6 p3, SkMel28 p3, SkMel2 p3). **(A)** Representative images of invasiveness through Matrigel-coated filters of A375M6, SkMel28, SkMel2 melanoma cells (control vs*.* p3). Scale bar: 200 μm. **(B)** mRNA expression evaluated by quantitative real-time PCR of the EMT markers N-cadherin, Vimentin, ZEB1 and SNAIL1 of A375M6, SkMel28, SkMel2 melanoma cells (control vs*.* p3). **(C)** Western blot analysis of N-Cadherin and IkB of A375M6, SkMel28, SkMel2 melanoma cells (control vs*.* p3). Levels of N-Cadherin and IkB were quantified by densitometric analysis and a corresponding histogram was constructed as relative to α-tubulin. Representative Western blot panels on the left. **(D)** mRNA expression evaluated by quantitative real-time PCR of the stemness markers SOX2, OCT3/4, KFL4 and ALDH1A1. Values presented are mean ± SEM of three independent experiments *P < 0.05 compared with control cells.

Since *anoikis* evasion is often associated with the acquisition of invasive and metastatic properties driven by EMT (Cao et al., 2016), we evaluated cell invasiveness and the expression of some EMT markers, including the expression of IkB, the inhibitor of the nuclear factor-κB (NF-κB), a transcription factor required for the induction of the EMT and involved in regulating anoikis (Lin et al., 2011). p3 anoikis-resistant melanoma cells showed a higher level of the EMT markers N-Cadherin, Vimentin, Zeb1, and SNAIL1 (Figure 2B), associated with high invasive ability (Figure 2A) and low IKB expression (Figure 2C), which indicates NF-kB activation. Moreover, since a stem-like phenotype sustains cancer cell survival after detachment from ECM, we evaluated the expression of SOX2, OCT3/4, KFL4 and ALDH1 stemness markers and we found that p3 anoikis-resistant cells show a tendency to increase the expression of these markers, especially ALDH1 (Figure 2D). This finding highlights that melanoma cell resistance in a detached condition needs a transition to a mesenchymal phenotype with some aspect of stemness.

**FIGURE 3:**
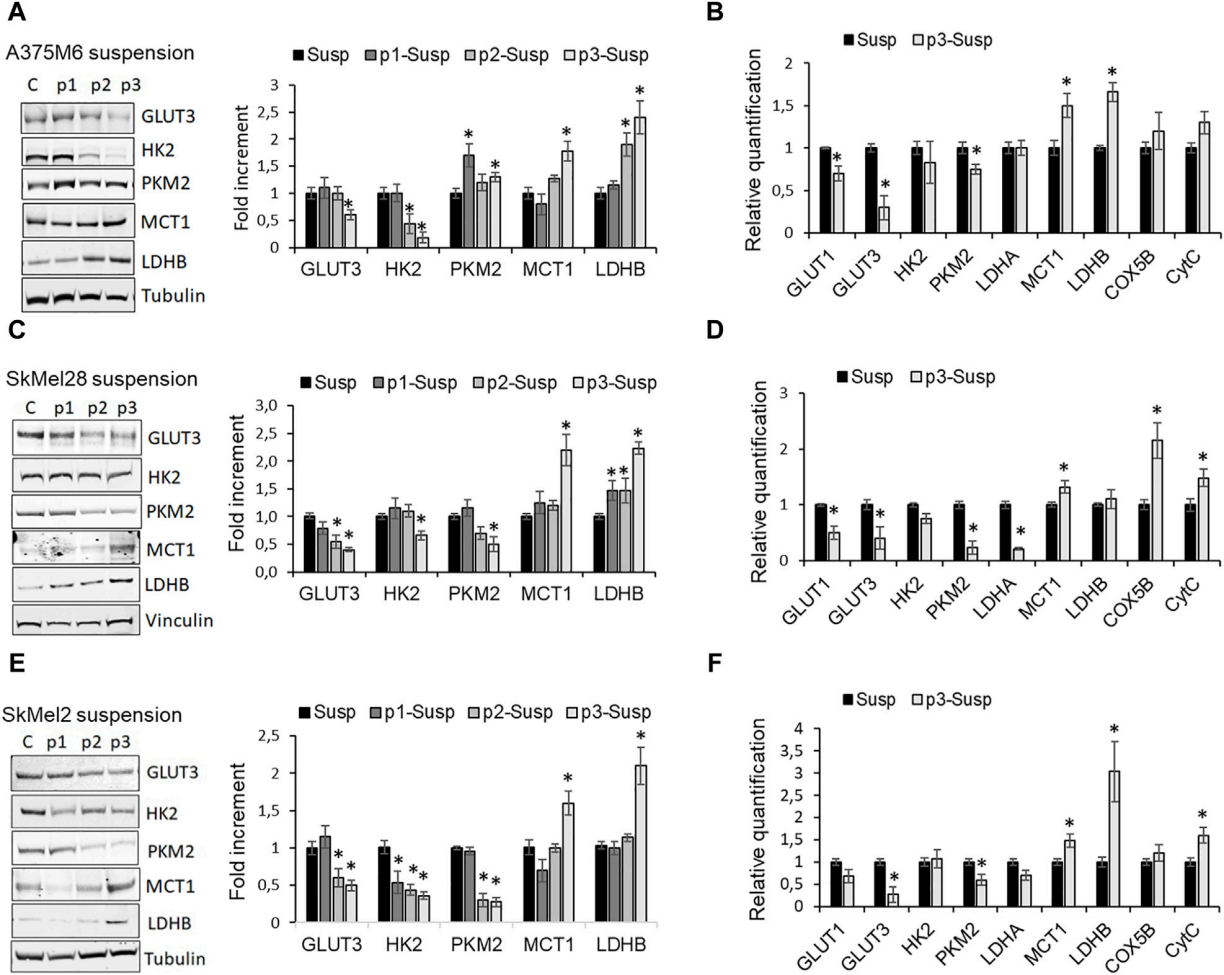
Metabolic profile of p3 melanoma cells: evaluation of the expression of glycolytic and oxidative markers. Western blot analysis of GLUT3, HK2, PKM2, MCT1, LDHB of A375M6 **(A)**, SkMel28 **(C)**, SkMel2 **(E)** melanoma cells in suspension after 1-4 consecutive rocking exposures (susp, p1 susp, p2 susp, p3 susp). Representative Western blot panels on the left. Protein levels were quantified by densitometric analysis and a corresponding histogram was constructed relative to α-tubulin. mRNA expression evaluated by quantitative real-time PCR of the glycolytic markers GLUT1, GLUT3, HK2, PKM2, LDHA, and of the oxidative markers MCT1, LDHB, COX5B, CytC of A375M6 **(B)**, SkMel28 **(D)**, SkMel2 **(F)** melanoma cells in suspension after 1 (Susp) or 4 (p3-susp) rocking exposures. Values presented are mean ± SEM of three independent experiments *P < 0.05 compared with control cells.

The primary methods for detecting CTCs are based on their physical and/or epithelial characteristics. However, the use of functional markers, such as altered metabolism, remains a subject of ongoing debate. Thus, we investigated the metabolic profile of *anoikis*-resistant cells as our CTC model, using dynamic and molecular approaches. To perform this aim, the p3 cells placed again in suspension in rocking condition for 24 h (p3-S) were compared for protein expression of glycolytic and oxidative markers with cells recovered at the end of each previous rocking step (S, p1-S, p2-S), before the re-attachment in culture dishes. We found that in all melanoma cell lines, the protein expression of the glycolytic markers glucose transporter (GLUT) 3, mediating glucose uptake from the extracellular environment, hexokinase (HK) 2, a critical enzyme catalyzing the first rate-limiting step of glycolysis and pyruvate kinase M2 (PKM2), gradually decreases as the steps in rocking condition progress from S to p3-S (Figures 3A, C, E). We also compared mRNA expression of glycolytic markers in p3-S melanoma cells respect to S melanoma cells and we confirmed that in *anoikis*-resistant cells the expression of GLUT1, GLUT3, HK2, PKM2, and LDHA significantly reduced (Figures 3B, D, F).

Conversely, the protein expression of the oxidative markers monocarboxylate transporter (MCT) 1, which mediates lactate uptake and lactate dehydrogenase (LDH) B, which catalyzes the conversion of lactate to pyruvate, progressively increases from S to p3-S (Figures 3A, C, E), as mRNA expression of MCT1, LDHB, Cytochrome C Oxidase Subunit 5B (COX5B) and Cytochrome C (CytC) (Figures 3B, D, F).

**FIGURE 4:**
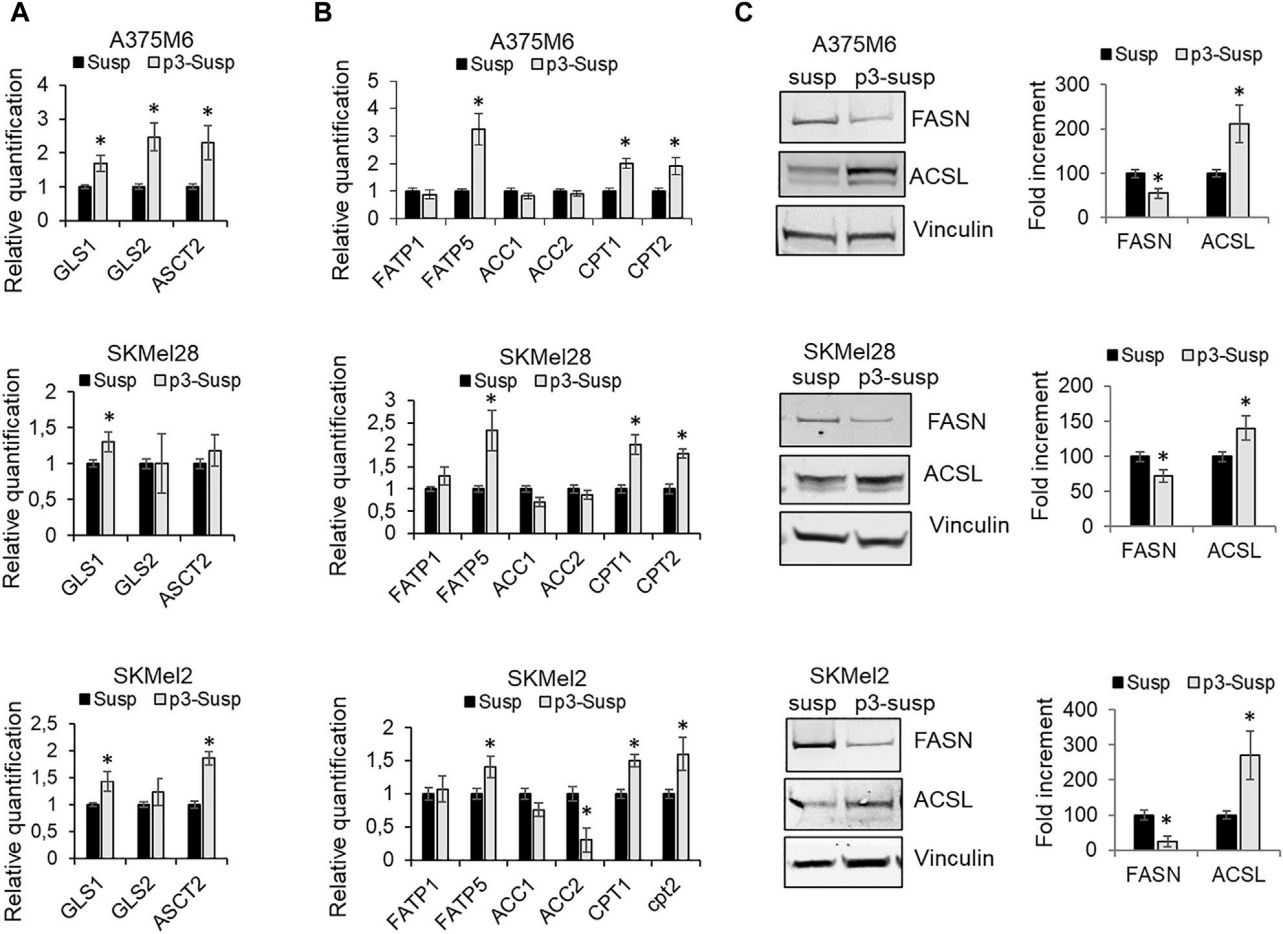
Metabolic profile of p3 melanoma cells: evaluation of markers of glutamine and fatty acid metabolism. mRNA expression evaluated by quantitative real-time PCR of **(A)** some markers of glutamine metabolism, GLS1, GLS2 ASCT2 and **(B)** markers of fatty acid metabolism, such as FATP1, FATP5, ACC1, ACC2, CPT1, CPT2 of A375M6, SkMel28, SkMel2 melanoma cells in suspension after 1 (Susp) or 4 (p3-susp) rocking exposures. **(C)** Western blot analysis of FASN and ACSL of A375M6, SkMel28, SkMel2 melanoma cells in suspension after 1 (Susp) or 4 (p3-susp) rocking exposures. Representative Western blot panels on the left. Protein levels were quantified by densitometric analysis and a corresponding histogram was constructed relative to vinculin. Values presented are mean ± SEM of three independent experiments *P < 0.05 compared with control cells.

A further source of carbon and nitrogen for both catabolic and anabolic demands is glutamine. The importance of glutamine lies in its being converted to glutamate and then to α-ketoglutarate (α-KG), a catabolic process known as glutaminolysis. α-KG enters the tricarboxylic acid (TCA) cycle, referred to as anaplerosis, not only for the generation of ATP via oxidative phosphorylation, but also for the production of acetyl-coA as a critical precursor for lipid and nucleotide synthesis. Many malignant tumor cells always display glutamine addiction. Alanine, serine, cysteine-preferring transporter 2 (ASCT2; SLC1A5) mediates the uptake of glutamine, while glutaminase (GLS), identified in two forms, GLS1 and GLS2, is the metabolic enzyme that catalyzes the first step in mitochondrial glutaminolysis. We observed that GLS mRNA expression, in particular the GLS1 isoform, and ASCT2 expression were increased in p3-S respect to S cells (Figure 4A).

However, to maintain a malignant phenotype, cancer cells exposed to an unfavorable tumor microenvironment need additional bioenergetic pathways, such as the mitochondrial fatty acid β-oxidation (FAO) (Qu et al., 2016). mRNA expression analysis of lipid metabolic markers revealed that p3-S melanoma cells express higher levels of fatty acid transporters FATP1 and FATP5 and comparable (or lower) levels of acetyl-CoA carboxylases 1 and 2 (ACC1, ACC2) when compared with S populations. ACCs are enzymes that catalyze the carboxylation of acetyl-CoA to produce malonyl-CoA. ACC1 localizes in the cytosol and acts as the first rate-limiting enzyme in the *de novo* fatty acid synthesis pathway, ACC2 localizes on the outer membrane of mitochondria and produces malonyl-CoA to inhibit carnitine palmitoyl-transferase 1 (CPT1) involved in the β-oxidation of fatty acids. P3-S melanoma cells express higher levels with respect to S melanoma cells of carnitine palmitoyl-transferases CPT1 and CPT2, which are mitochondrial enzymes responsible for the formation of acylcarnitines by catalyzing the transfer of the acyl group of a long-chain fatty acyl-CoA from coenzyme A to l-carnitine (Figure 4B). We also evaluated by Western blot analysis the protein expression of the multi-enzyme protein Fatty acid synthase (FASN), which catalyzes the *de novo* synthesis of palmitate, and of the intrinsic membrane proteins Long-chain fatty acyl-CoA synthetases (ACSLs), which catalyze the conversion of fatty acids to their corresponding fatty acyl-CoAs (Figure 4C). ACSL1 has a specific function in directing the metabolic partitioning of FAs toward β-oxidation in adipocytes (Ellis et al., 2010). In agreement with our previously described results, we found that p3-S cells show low FASN expression and high ACSL levels. On the whole, our results suggest that *anoikis*-resistant melanoma sub-populations increase glutamine and fatty acid uptake and fatty acid beta-oxidation revealing a transition from a glycolytic to a more oxidative phenotype based on glutamine and fatty acid substrates.

### 3.2 Effect of metabolic drugs on *anoikis*-resistant melanoma cells

**FIGURE 5:**
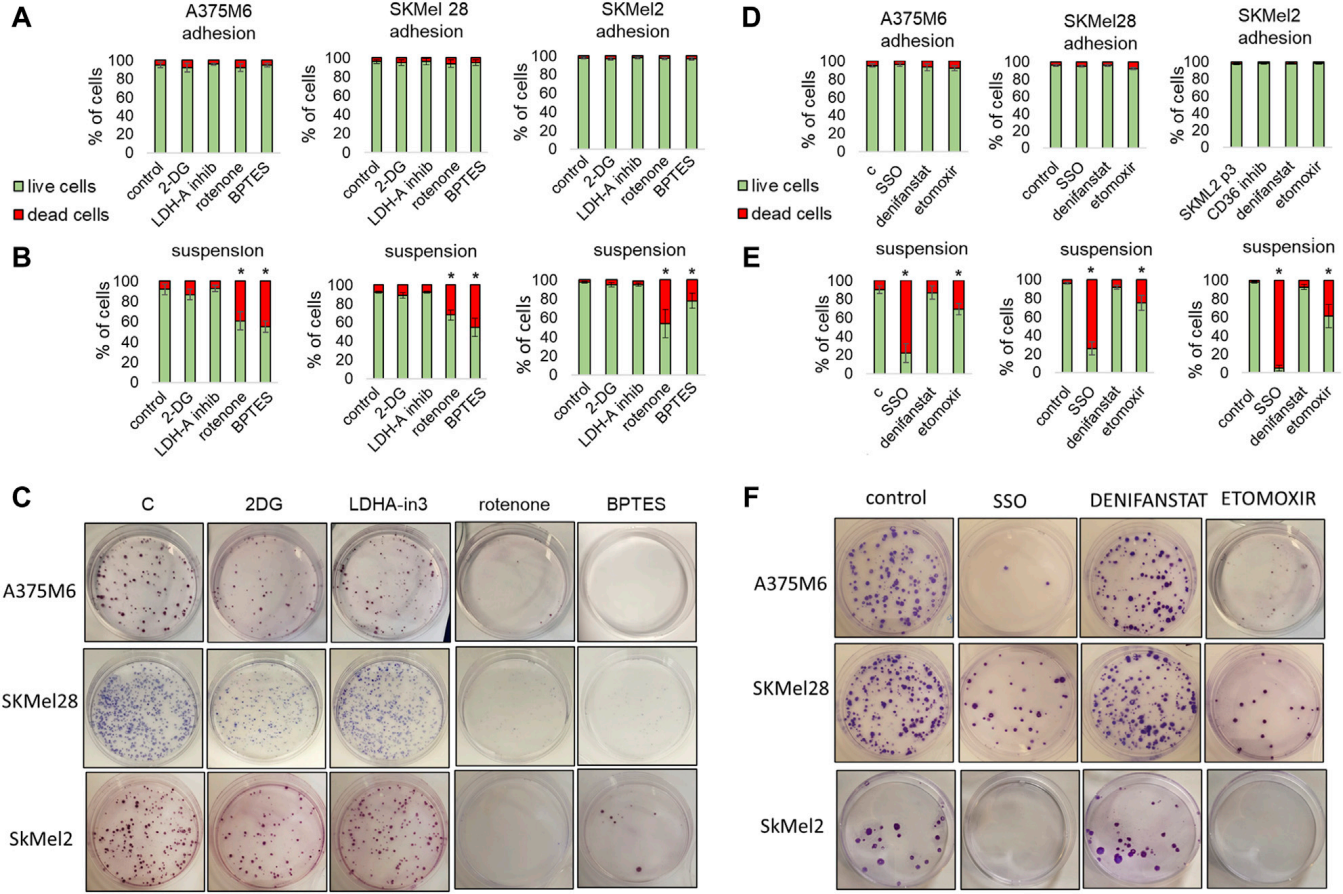
Effect of metabolic inhibitors on melanoma cell survival under non-adherent condition. A375M6, SkMel28, SkMel2 p3 melanoma cells under adherent (adhesion) **(A,D)** or rocking conditions (Suspension) **(B,C,E,F)** were treated for 24 h with 2DG (20 mM), LDH-in-3 (50 μM), rotenone (1 μM), BPTES (10 μM) **(A–C)**, SSO (50 μM), denifanstat (10 μM) or etomoxir (50 μM) **(D,E,F)**. After the treatment cells were analyzed for cell viability, evaluated by LIVE/DEAD Fixable Violet Dead Cell Staining **(A,B,D,E)** or for clonogenic efficiency **(C,F)**. Data are expressed as mean ± SEM of at least three independent experiments; images are representative of experiments performed in triplicate. *P < 0.05 compared with control cells.

We treated p3 melanoma cells with different metabolic inhibitors during their incubation in rocking conditions to understand whether *anoikis*-resistant melanoma cells could be targetable with specific metabolic drugs. We first used glycolytic and oxidative inhibitors such as 2-Deoxy-D-Glucose (2DG), a glucose analog inhibiting HK2; LDH-IN-3, a potent noncompetitive LDHA inhibitor; rotenone, a mitochondrial respiratory chain complex I inhibitor; BPTES, a glutaminase inhibitor. At the end of the incubation in rocking condition, we analyzed the viability of the cells by flow cytometry and the cloning efficiency by colony formation assay. We found that while none of the treatments had any effects on the viability of cells grown in adhesion (viability always above 92%) (Figure 5A; cell viability was significantly affected by specific drugs when the treatment was carried out during the rocking condition (Figure 5B; representative flow cytometric plots in Supplementary Figures S4–S6). In particular rotenone and BPTES caused more than 25% cell death. The colony formation assay corroborated the results previously described, albeit with a more pronounced effect. Cells treated with 2DG showed a weak reduction in the number of colonies, which was significant only in SKMel28, while LDHA-IN-3 showed no significant effect in all three cell lines used. Rotenone and BPTES almost completely blocked colony formation in all the different cell lines (Figure 5C). Finally, we tested the effect of fatty acid metabolism inhibitors: sulfosuccinimidyl oleate (SSO), a long-chain fatty acid that inhibits fatty acid transport into cells; denifanstat, a potent Fatty Acid Synthase (FASN) inhibitor; etomoxir, which blocks fatty acid oxidation through CPT-1a inhibition. 24-h treatment with these drugs did not affect the viability of adherent cells (Figure 5D). When these drugs were used on suspended cells, we observed that SSO caused a mortality of more than 80%, etomoxir mortality of more than 25%, while the cells treated with denifanstat showed a mortality comparable to control cells (about 2%–5%) (Figure 5E; representative flow cytometric plots in Supplementary Figures S4–S6). When we analyzed the colony formation ability, we found that etomoxir and sulfosuccinimidyl oleate significantly reduced colony number, while denifanstat showed no effect (Figure 5F).

These findings confirm that the *anoikis*-resistant melanoma cells require glutamine and fatty acid metabolic rewiring.

### 3.3 Effect of the adhesive reattachment of anoikis-resistant melanoma cells on their metabolic rewiring

**FIGURE 6:**
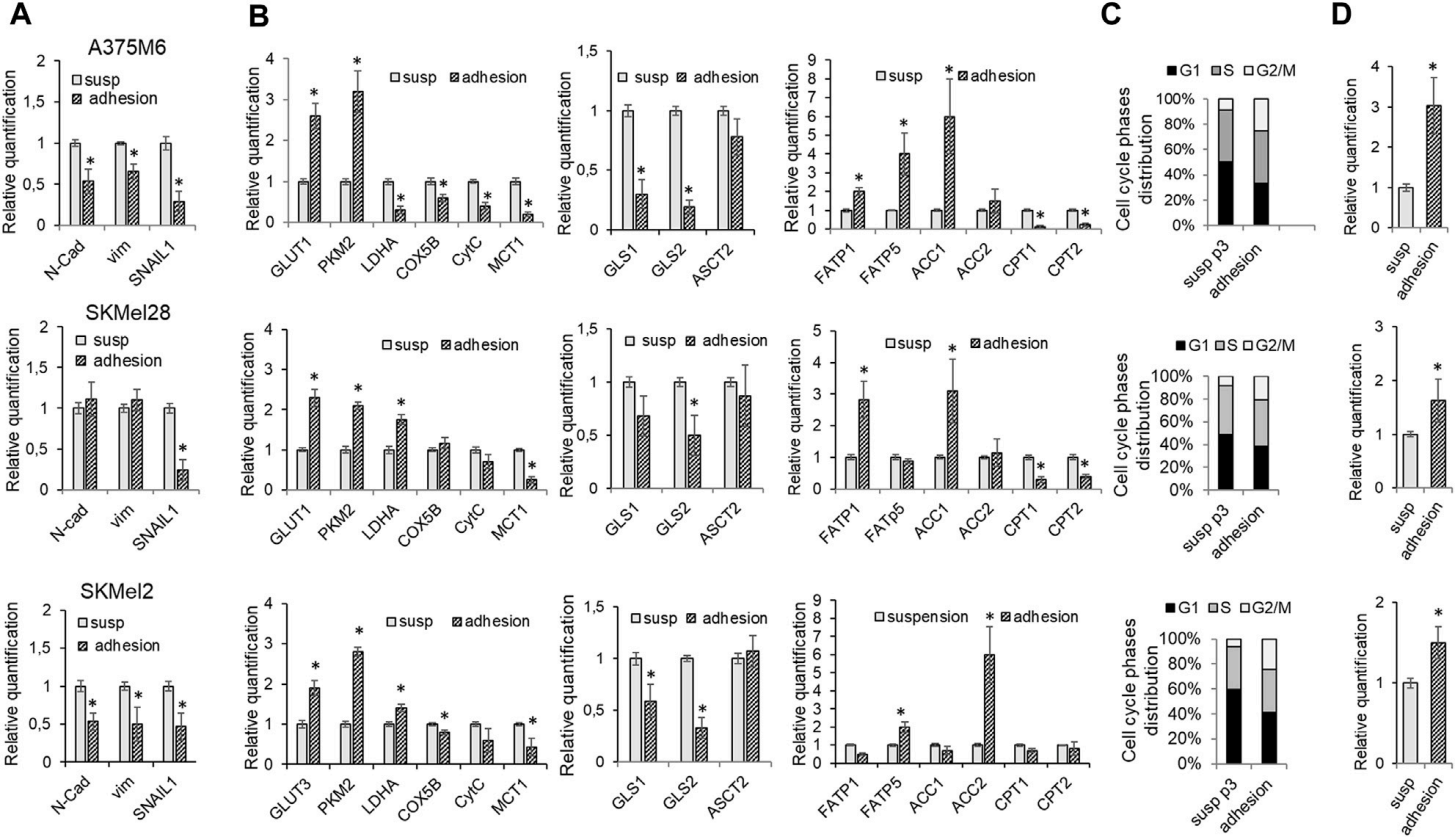
Effect of the adhesive re-attachment of *anoikis*-resistant melanoma cells on their metabolic rewiring. A375M6, SkMel28, SkMel2 p3 melanoma cells were subjected to a rocking exposure in suspension (susp) and then allowed to re-grow in adherent plastic dishes (adhesion). mRNA expression evaluated by quantitative real-time PCR of: **(A)** the EMT markers N-cadherin, Vimentin and SNAIL1, **(B)** the glycolytic markers GLUT1, PKM2, LDHA; the oxidative markers COX5B, CytC, MCT1; the markers of glutamine metabolism GLS1, GLS2, ASCT2 and of fatty acid metabolism FATP1, FATP5, ACC1, ACC2, CPT1, CPT2. **(C)** Cell cycle analysis and **(D)** mRNA expression evaluated by quantitative real-time PCR of Cyclin **(D)**. Values presented are mean ± SEM of three independent experiments *P < 0.05 compared with control cells.

When p3-S melanoma cells were allowed to re-grow in adherent plastic dishes, cells reconverted their EMT to MET, as indicated by the reduction in the expression of EMT markers N-Cadherin, Vimentin, and SNAIL1 shown in Figure 6A. We found that these cells also reconvert their metabolic attitude to aerobic glycolysis. Indeed, the expression of the glycolytic markers GLUT1, PKM2, and LDHA was increased, while the expression of the oxidative markers COX5B, CytC, and MCT1 was reduced, compared to parental suspended cells (Figure 6B). These changes are accompanied by a reduction in GLS1 and GLS2 expression (Figure 6B). Moreover, in this setting, melanoma cells express high levels of ACC1 and ACC2, as does the expression of FATP1 and FATP5, while CPT1 and CPT2 are reduced (Figure 6B), justifying a lipid synthesis to be used for proliferation or energy storage in lipid droplets. We performed the cell cycle analysis of *anoikis*-resistant melanoma cells and we found a reduction in the percentage of cells in the G0-G1 phase with an increase in cells in the G2/M phase in all three cell lines, suggesting a strong aid to enter the cell cycle and to initiate cell division (Figure 6C; Flow Cytometry representative histograms in Supplementary Figure S7). The increased expression in re-adapted cells of cyclin D (Figure 6D), a mitogenic sensor able to integrate extracellular signals and cell cycle progression, underlines the association, in these cells, of a proliferative with the regained glycolytic phenotype. These findings further indicate how *anoikis*-resistant melanoma cells, despite three consecutive selections in rocking conditions, are readily able to reconvert their phenotype, indicating a maintained plasticity. This ability may represent a sign of particular importance when CTCs undergo MET and colonize the new organ microenvironment.

### 3.4 Comparison of bioenergetic phenotype between adherent cells and anoikis-resistant tumor cell subpopulations

**FIGURE 7:**
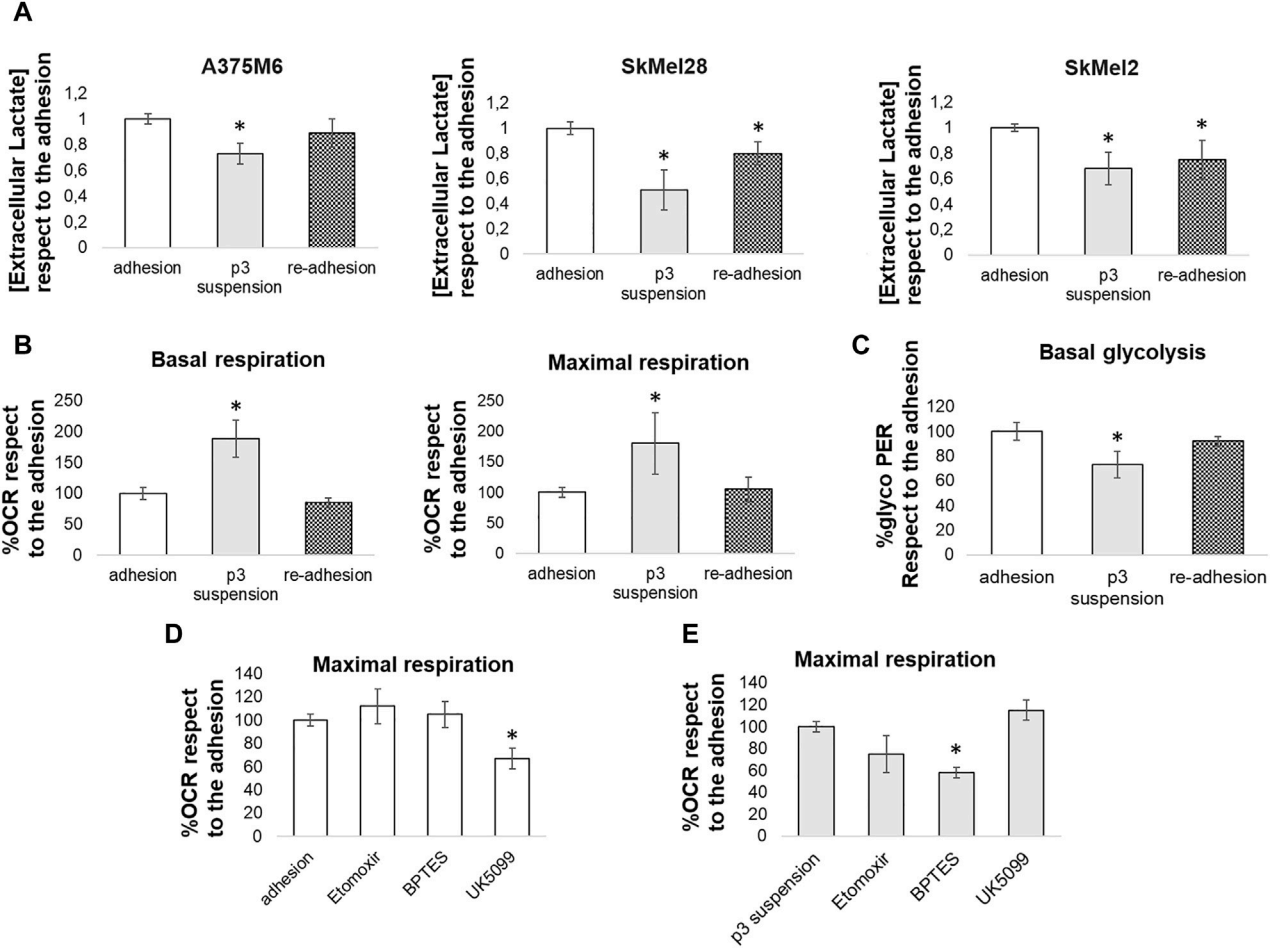
Comparison between the energy metabolism of adherent cells and *anoikis*-resistant melanoma cells. **(A)** Extracellular lactate levels were compared between melanoma cells (A375M6, SkMel28, SkMel2) grown in adherent conditions (adhesion), p3 cells subjected to a rocking exposure (p3 suspension) or p3 cells allowed to re-grow in adherent plastic dishes (re-adhesion). Adherent A375M6 cells, p3 A375M6 cells subjected to a rocking exposure or p3 A375M6 cells allowed to re-grow in adherent conditions were subjected to the Seahorse XF Mitochondrial stress test or to the glycolytic rate assay to evaluate oxygen consumption rate (OCR) and glycolytic proton efflux rate (glycoPER) and to determine basal and maximal respiration **(B)** and basal glycolysis **(C)**. The Seahorse XF substrate oxidation stress test was performed to determine the specific substrates oxidated by adherent A375M6 cells **(D)** and p3 A375M6 cells subjected to a rocking exposure **(E)**. Changes in OCR were evaluated after the injection of the indicated inhibitors: UK5099 (glucose oxidation inhibitor); etomoxir (fatty acid oxidation inhibitor); BPTES (glutamine oxidation inhibitor). Values presented are mean ± SEM of three independent experiments *P < 0.05 compared with control cells.

To confirm the different energy pathways used by adherent cells and *anoikis*-resistant cells, we first analyzed the extracellular lactate concentrations in media conditioned by control cells, p3 cells grown in rocking conditions, and p3 cells after re-adhesion. The measures of extracellular lactate levels confirmed the glycolytic metabolic profile of adherent cells (both control cells and p3 after re-adhesion), able to produce a high quantity of lactate, while p3 cells in suspension showed a lower level of extracellular lactate, in any cell lines used (Figure 7A).

The real-time measurement of cellular metabolism using the Seahorse XF96 Extracellular Flux Analyzer allowed us to analyze the Oxygen Consumption Rate (OCR) and the Proton Efflux Rate of A375M6 control cells (grown in adhesion), p3 cells grown in rocking conditions, and p3 cells after re-adhesion. The p3 population in suspension showed a significant increase in both basal and maximal respiration levels compared to adherent cells (Figure 7B; representative time-course traces of OCR in Supplementary Figure S8A) and a significant reduction in basal glycolysis (Figure 7C; representative time-course traces of Proton Efflux Rate in Supplementary Figure S8B). Control A375M6 melanoma cells showed higher levels of glycolysis and lower levels of OCR (Figures 7B, C).

To determine the specific substrates oxidated by cells grown in adherent conditions or p3 cells after 24 h in rocking conditions, we performed the substrate oxidation stress tests using the inhibitors specific for the three primary substrates that fuel the mitochondria: long-chain fatty acids (etomoxir), glucose/pyruvate (UK5099), and glutamine (BPTES). We found that the OCR of control adherent cells was significantly inhibited by UK5099 (Figure 7D; representative time-course traces of OCR in Supplementary Figure S8C), which blocks the use of glucose/pyruvate for respiration by inhibiting the mitochondrial pyruvate carrier. On the contrary, the OCR of p3 cells in suspension did not change after UK5099 treatment but was significantly inhibited by BPTES, a GLS inhibitor, and partially reduced by etomoxir, a CPT inhibitor (Figure 7E; representative time-course traces of OCR in Supplementary Figure S8C).

These results confirmed that cells in non-adherent conditions undergo a metabolic reprogramming changing the substrate used for respiration from glucose to fatty acids and glutamine.

### 3.5 Effect of metabolic drugs on non-melanoma cells exposed to a non-adherent condition

**FIGURE 8:**
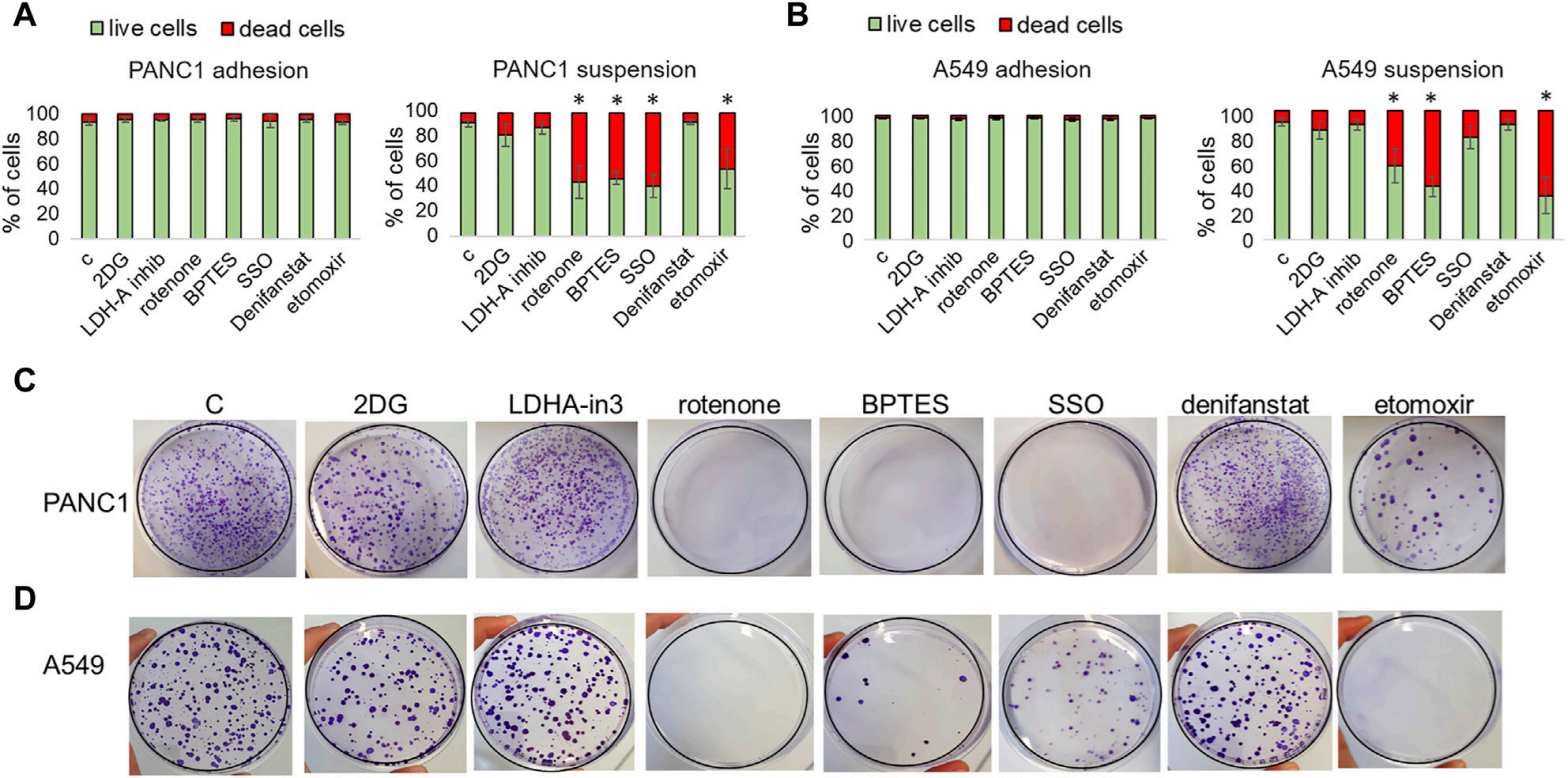
Effect of metabolic drugs on non-melanoma cells exposed to a non-adherent condition. PANC1 **(A,C)** and A549 **(B,D)** cells under adherent (adhesion) or rocking conditions (suspension) were treated for 24 h with 2DG (20 mM), LDH-in-3 (50 μM), rotenone (1 μM), BPTES (10 μM), SSO (50 μM), denifanstat (10 μM) or etomoxir (50 μM). After the treatment cells were analyzed for cell viability, evaluated by LIVE/DEAD Fixable Violet Dead Cell Staining **(A,B)** or for clonogenic efficiency **(C,D)**. Data are expressed as mean ± SEM of at least three independent experiments; images are representative of experiments performed in triplicate. *P < 0.05 compared with control cells.

We extended our investigation to tumor cells of a different histotype. We tested some of the metabolic drugs on PANC1 (a human pancreatic cancer cell line) and A549 cells (an adenocarcinoma human alveolar cell line) exposed for 24 h to a rocking condition. At the end of the incubation in rocking condition, we analyzed cell viability by flow cytometry and the cloning efficiency by colony formation assay. We found that none of the treatments had any effects on the viability of cells grown in adhesion (viability always above 94%), while viability was significantly affected by the inhibitors of glutaminase (BPTES), fatty acid uptake (SSO), beta-oxidation (etomoxir) when the treatment was carried out during the rocking condition (Figures 8A, B; representative flow cytometric plots in Supplementary Figures S9, S10). BPTES, SSO, etomoxir, and Rotenone resulted able to suppress colony efficiency (Figures 8C, D), as it has been previously observed using the p3 anoikis-resistant melanoma cells. It is possible that a rapid reconversion to an oxidative metabolism using some available fuels, such as glutamine and fatty acids, represents a general transition of tumor metabolism during the loss of adhesiveness.

## 4 Discussion

Understanding tumor metabolism and its reprogramming when tumor cells adapt to a changing microenvironment is fundamental for developing innovative therapies. Indeed, cell energetic metabolism is deeply interconnected with and influenced by the surrounding environment. Tumor cells need to adapt their metabolism in response to changes in oxygen levels, such as moving from aerobic to anaerobic glycolysis or compensating for glucose deprivation by utilizing alternative nutrients, thereby switching from glycolysis to oxidative metabolism (Boroughs and DeBerardinis, 2015). Metabolic reprogramming is now considered a hallmark of cancer (Hanahan and Weinberg, 2011). Particularly, tumor cells need to adapt their metabolic phenotype during metastatic dissemination in the bloodstream, when are deprived of matrix attachment and subjected to various physical and physiological stresses including nutrient deprivation. This special condition deserves a transition of tumor cells to a less proliferative state and metabolic reprogramming to overcome the programmed cell death, known as “*anoikis*.” Consequently, the metabolic reprogramming of *anoikis*-resistant tumor cells has emerged as a focal point in understanding tumor cell dissemination (Adeshakin et al., 2021).

We investigated the metabolic reprogramming of some selected high *anoikis*-resistant melanoma cells, e.g., A375M6, SKMel2, and SKMel28 cells. Previous investigations addressed the major mechanisms of the *anoikis* resistance of epithelial-derived tumor cells, whereas less attention is devoted to melanoma cells, highly migratory cells of neuroectodermal origin characterized by a high inter- and intra-tumor heterogeneity. This heterogeneity leads to different plastic phenotypes that play an important role in resistance. Our study’s model is represented by melanoma cells selected for a high *anoikis* resistance, obtained through three consecutive exposures to a 24-h rocking condition, resulting in the selected subpopulation, referred to as the p3 cells. The procedure for the high *anoikis*-resistant p3 cell selection represents our attempt to mimic the rapid selection operated by the bloodstream on tumor cells that leave the primary tumor and enter into the circulation. It is known that most tumor cells that enter the bloodstream disappear in 24 h (Fidler, 1975; Kowalik et al., 2017). We confirmed that the p3-selected melanoma cells when transferred to agarose-coated dishes, a further condition where the adhesion is prevented, show a high colony formation ability. Furthermore, when the p3-selected cells were once again exposed to a rocking condition, they exhibited higher viability compared to parental cells and a significantly enhanced ability to form colonies. These results reveal the ability of p3-selected cells to sustain a certain level of *anoikis* resistance and generate new colonies under adhesive conditions. Although p3-selected melanoma cells maintain a certain heterogeneity, most cells show a transition phenotype toward a more mesenchymal-like type, with high *in vitro* invasiveness. Indeed, the p3-selected melanoma cells express the most characteristic EMT markers (see Chiarugi and Giannoni, 2008) and a high ALDH1 among the stem cell markers. ALDH1 is a detoxifying enzyme expressed by some epithelial cancer stem cells able to metabolize a wide variety of intracellular aldehydes providing resistance to alkylating chemicals as cyclophosphamide (Deng et al., 2010; Luo et al., 2012). These findings reveal that the p3-selected subpopulations, obtained for three successive exposures to a non-adhesive condition, express important features of resistance, invasiveness, and stemness associated with the ability to survive in suspension. The significance of EMT markers and a partial stem cell-like phenotype of p3-selected melanoma cells may render these findings a possible resource for CTC diagnosis. *Anoikis* represents the major challenge met by cells of a solid tumor during their diffusion into the circulation. The ability to resist *anoikis* also requires a metabolic flexibility able to sustain survival even during a nutrient limitation in a stress condition, such as blood turbulence, which might require a high energy expenditure: *anoiki*s-resistance and the metabolic adaptation of tumor cells as two sides of the same coin. We studied the metabolic profile of p3-selected melanoma cells placed again in suspension, to prevent any possible influence of mechanisms involved in extracellular anchorage. Indeed, at the secondary site, when metastatic cancer cells recover the attachment to a new extracellular substrate and find abundant nutrients, they may switch to a proliferative phenotype linked to a further metabolic change. We found that p3-selected melanoma cells still in suspension show a reduction of glycolytic markers from p1 to p3 subpopulations, at a mRNA, and protein level evaluation confirmed these changes. In parallel, the same cells express higher levels of oxidative phosphorylation markers suggesting a change of p3-selected cells from classical glycolytic phenotype to an oxidative metabolism. Glutamine is one of the most abundant amino acids in plasma and is important for ATP generation and “anaplerosis,” which is the process of supplying nitrogen sources for molecular building block synthesis (Kodama et al., 2020). Markers of glutamine metabolism expressed by p3-selected melanoma cells indicate a promoted uptake and catabolism of glutamine via GLS1, required for ATP synthesis. This finding suggests that p3-selected melanoma cells force the use of glutamine as a metabolic fuel instead of glucose. However, under stressed conditions, cancer cells may also adapt to the use of fatty acid metabolism, and fatty acid oxidation (β oxidation) provides more than twice as much ATP as carbohydrates and reduces reactive oxygen species producing NADH and FADH2 (Carracedo et al., 2013). Fatty acids may be acquired from the bloodstream as free fatty acids or within lipoproteins or can be mobilized from cytoplasmic lipid droplets. Malonyl-CoA synthesized by the activity of ACC is the key metabolite at the crossroads of biosynthesis and degradation of fatty acids and CPT is the rate-limiting step in FA oxidation. Markers of fatty acid metabolism of p3-selected melanoma cells indicate an enhanced uptake, activation, and catabolism of fatty acids in the presence of a reduced synthesis. Thus, p3 *anoikis*-resistant melanoma cells seem to switch to an energy-generating fatty acid oxidation metabolism and inhibition of fatty acid synthesis to save energy. The use of several metabolic inhibitors of glucose, glutamine, and fatty acid metabolism confirmed that p3-selected melanoma cells are addicted to the use of oxidative metabolism of glutamine and fatty acids. It is known that FAO induced in breast cancer cells promotes *anoikis* resistance and ALDH 1 activity (De Francesco EM et al., 2017; Bapat and Bitler, 2021), whereas targeting FAO stimulates *anoikis* of ovarian cancer cells (Sawyer et al., 2020).

Of particular interest, p3-selected melanoma cells when re-adapted to growth in an adhesive condition in culture dishes, switched back to a proliferative glycolytic phenotype and fatty acid synthesis, suggesting that although the p3 melanoma subpopulation enriched in *anoikis*-resistant cells express an enhanced oxidative metabolism when in suspension, they retain the ability to promptly reconvert to a glycolytic phenotype, appropriate for cell proliferation and mass expansion in a secondary organ.

This dynamic cell metabolic reprogramming was confirmed by the measurement of lactate production and through metabolic analysis performed using the Seahorse XF96 Extracellular Flux Analyzer, comparing adherent cells, p3 melanoma subpopulation and p3 cells re-adapted to growth in adherent conditions. p3 melanoma subpopulation showed high oxygen consumption rate levels, low proton efflux rate, accompanied with reduced levels of extracellular lactate, all results in agreement with the low expression of glycolytic markers shown by these cells. All these parameters go back to levels comparable to control adherent cells, when p3 cells re-adapt to growth in adherent conditions, confirming the ability of *anoikis*-resistant cells to reconvert to a glycolytic phenotype, under specific scenarios.

The Seahorse XF analysis of the specific substrates oxidated by p3 subpopulation or adherent cells confirmed that, while cells in adhesion exploit glucose, for both glycolysis and respiration, p3 cells mainly use glutamine and fatty acids.

After all, we found that the metabolic changes in the direction of the use of oxidative metabolism of glutamine and fatty acids also characterize some *anoikis*-resistant human pancreatic and alveolar cancer cells.

On the whole, our data suggest that melanoma cells able to survive in blood circulation may reprogram their glycolytic phenotype to oxidative phosphorylation of glutamine and fatty acids. These findings open up to organizing a complementary therapy for metastatic melanoma based on specific metabolic inhibitors.

## Data Availability

The raw data supporting the conclusions of this article will be made available by the authors, without undue reservation.
