# Supplementary material for: The critical role of glutamine and fatty acids in the metabolic reprogramming of anoikis-resistant melanoma cells
Source: Front Pharmacol. 2024 Aug 8;15:1422281. doi: 10.3389/fphar.2024.1422281 (PMC11338773; doi:10.3389/fphar.2024.1422281)
Supplement: Supplementary file 1 [file DataSheet1.PDF]

Supplementary material

The critical role of glutamine and fatty acids in the metabolic reprogramming of *anoikis*-resistant melanoma cells

Peppicelli S., Kersikla T., Menegazzi G., Andreucci E., Ruzzolini J., Nediani C., Bianchini F., Calorini L.

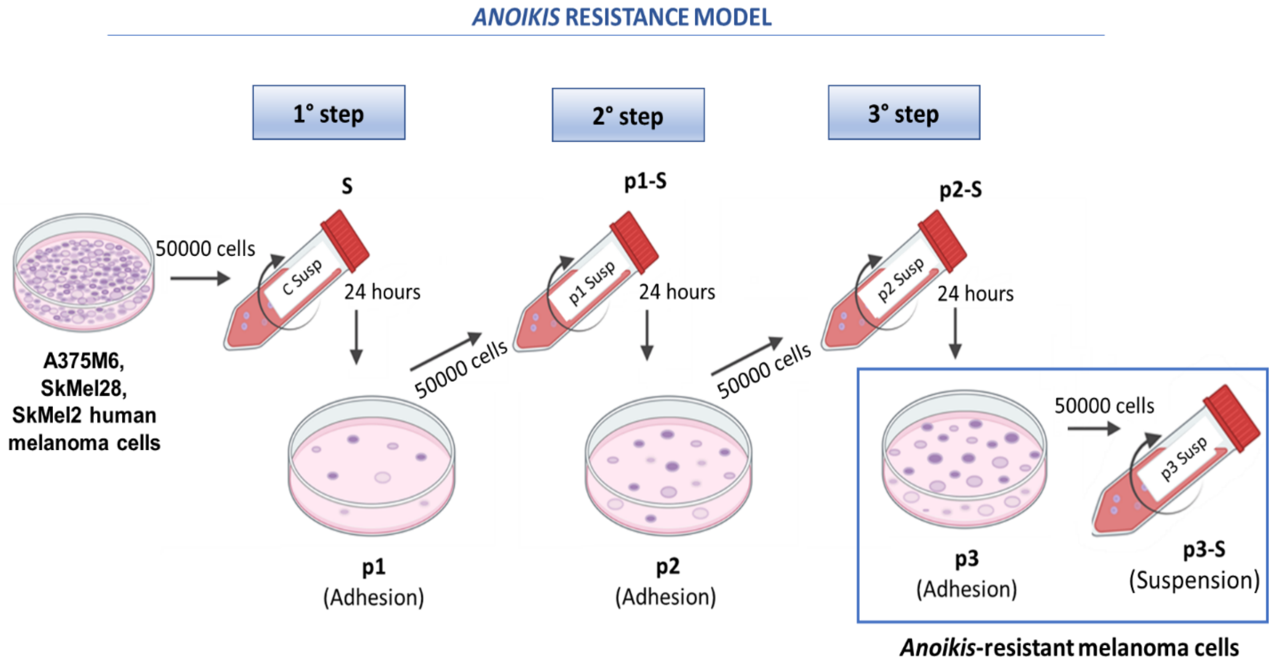

Figure S1- To select melanoma cells with a well-consolidated *anoik*is-resistant phenotype A375M6, SkMel28, and SkMel2 human melanoma cells were exposed several times to a loss of adherence condition. $5x10^4$ melanoma cells suspended in a growth factor-free DMEM/F12 medium, were placed in sterile non-adherent 50 ml tubes, that were shacked on a Mini rocker platform shaker (20° angle) for 24 hours, at 37°C. Cells were recovered and placed on an adherent plastic dish to grow until reaching an adequate number to be used to proceed with further rocking exposure. At the end of the third rocking exposure (p2-Suspension, p2-S), recovered cells were grown in an adherent plastic dish to obtain the p3-Adhesion population, which was tested for *anoikis* resistance.

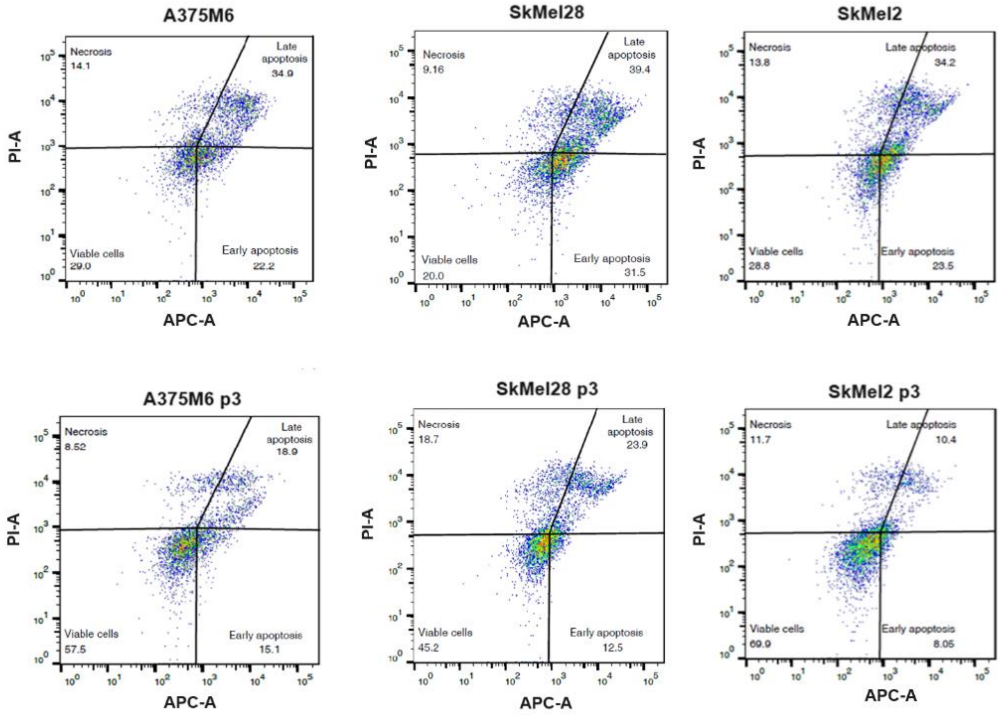

Figure S2- Representative cytograms from flow cytometry analysis of apoptotic cells revealed by Annexin V-APC staining of control or p3 A375M6, SkMel28, and SkMel2 cells after 24 h in rocking condition.

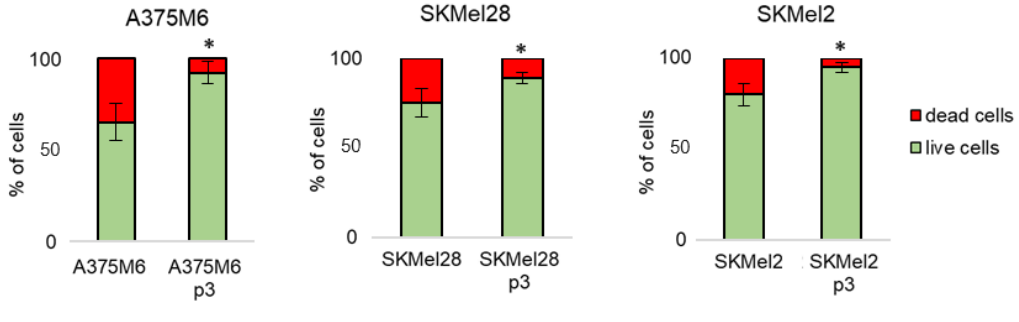

Figure S3- Cell viability, evaluated by LIVE/DEAD Fixable Violet Dead Cell Staining of melanoma cells (control vs p3) after exposure to 24 hours of rocking conditions. Values presented are mean ± SEM of three independent experiments. *P < 0.05 compared with control cells.

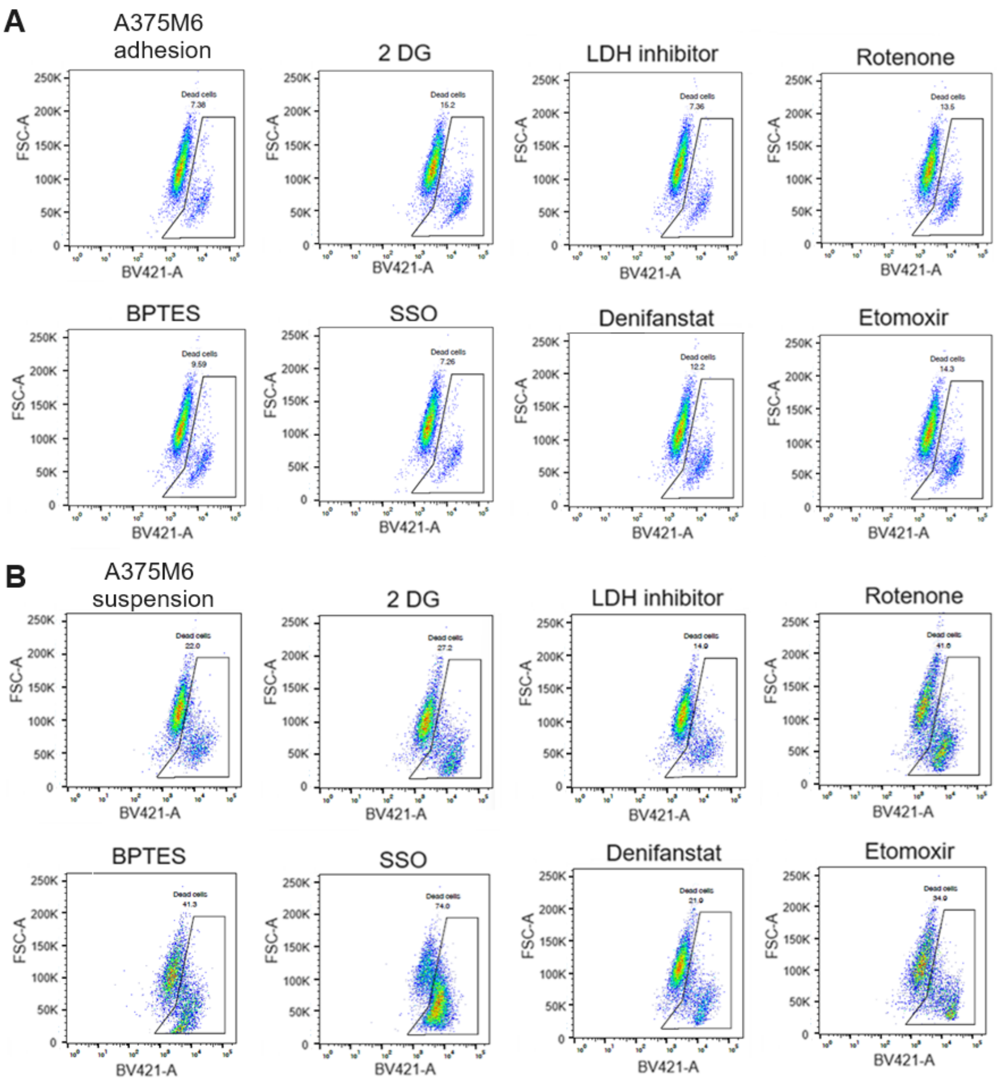

Figure S4- Representative plots of LIVE/DEAD Fixable Violet Dead Cell Staining of A375M6 melanoma cells under adherent (adhesion) (A) or rocking conditions (Suspension) (B), treated for 24 hours with 2DG, LDH-in-3, rotenone, BPTES, SSO, denifanstat or etomoxir.

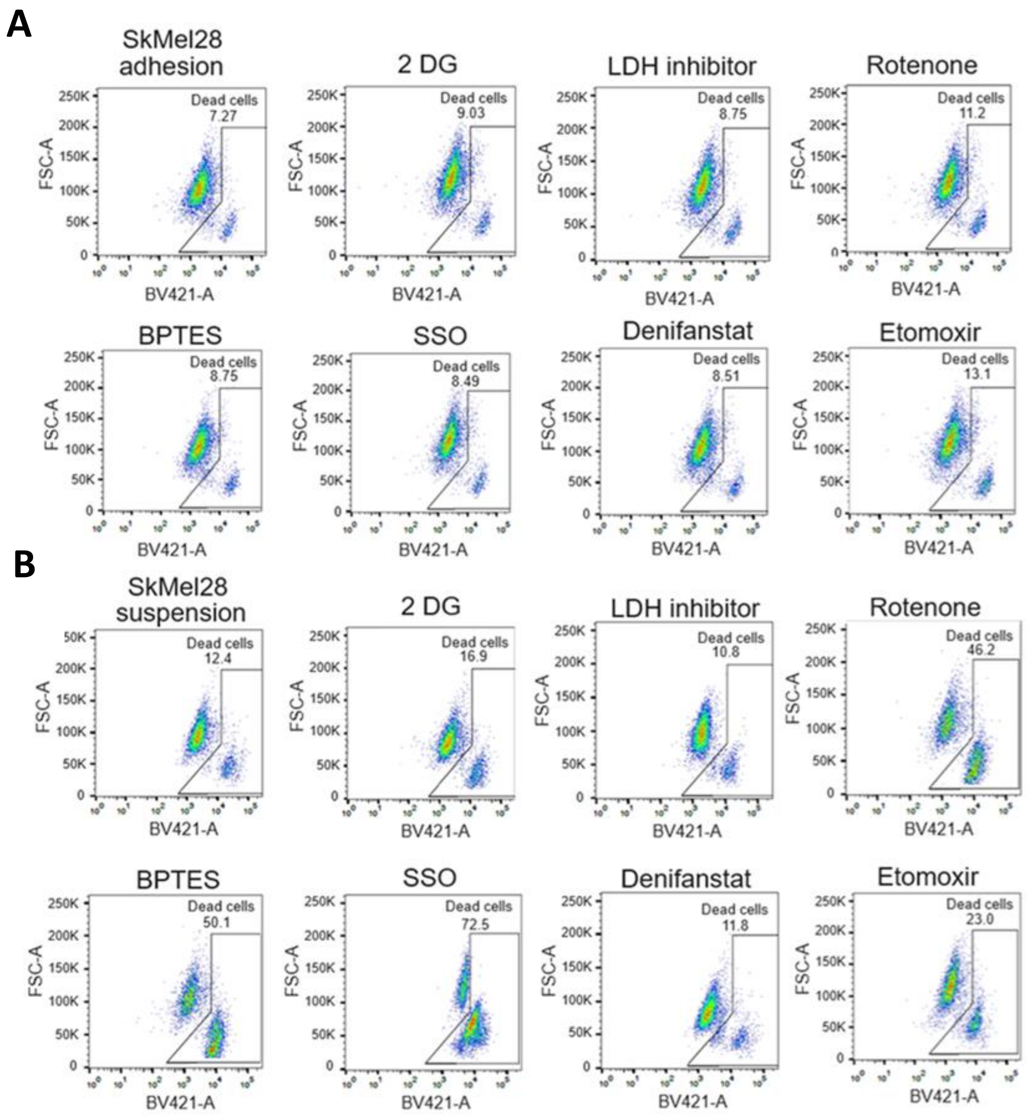

Figure S5- Representative plots of LIVE/DEAD Fixable Violet Dead Cell Staining of SkMel28 melanoma cells under adherent (adhesion) (A) or rocking conditions (Suspension) (B), treated for 24 hours with 2DG, LDH-in-3, rotenone, BPTES, SSO, denifanstat or etomoxir.

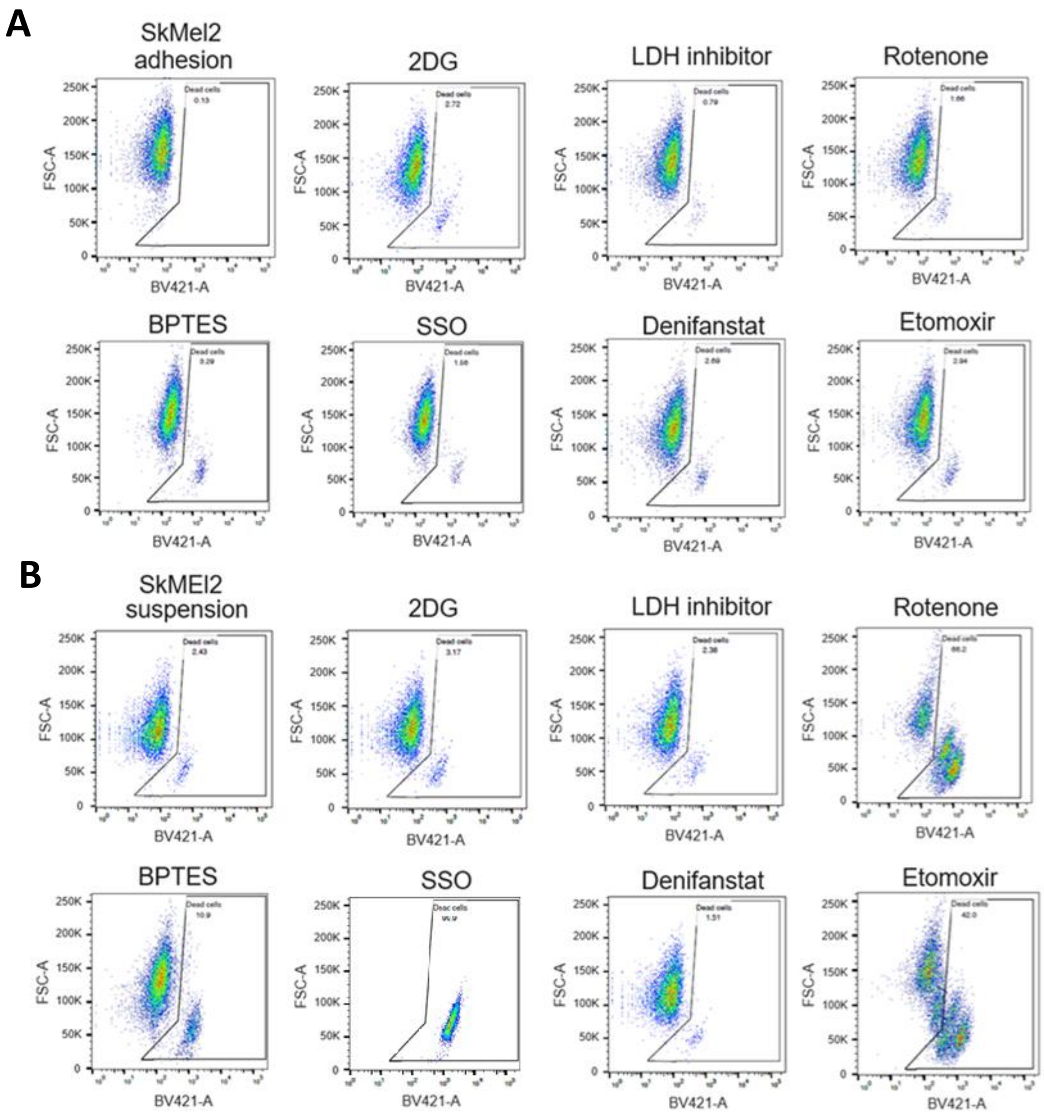

Figure S6- Representative plots of LIVE/DEAD Fixable Violet Dead Cell Staining of SkMel2 melanoma cells under adherent (adhesion) (A) or rocking conditions (Suspension) (B), treated for 24 hours with 2DG, LDH-in-3, rotenone, BPTES, SSO, denifanstat or etomoxir.

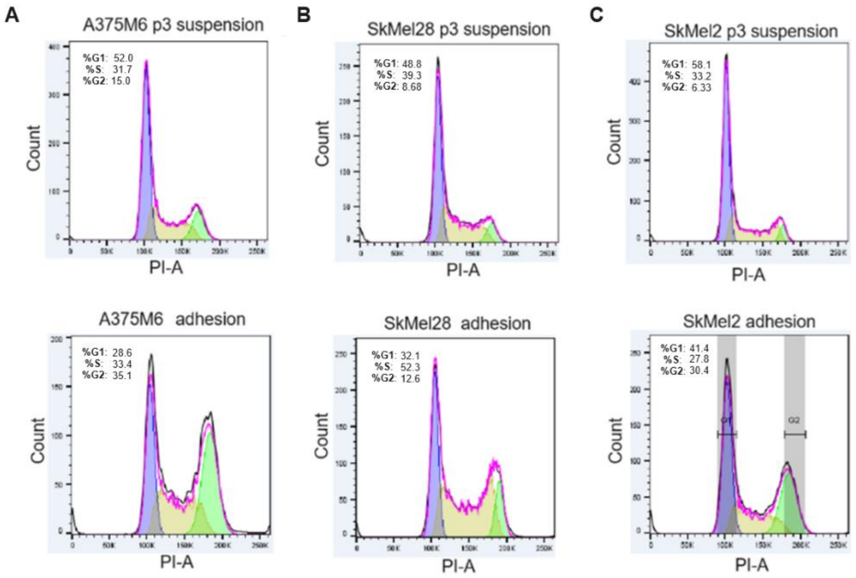

Figure S7- Representative histograms of cell cycle distribution performed by PI staining in A375M6 (A), SkMel28 (B), SkMel2 (C) p3-S populations after 24h in rocking conditions or after re-attachment to adherent plastic dishes.

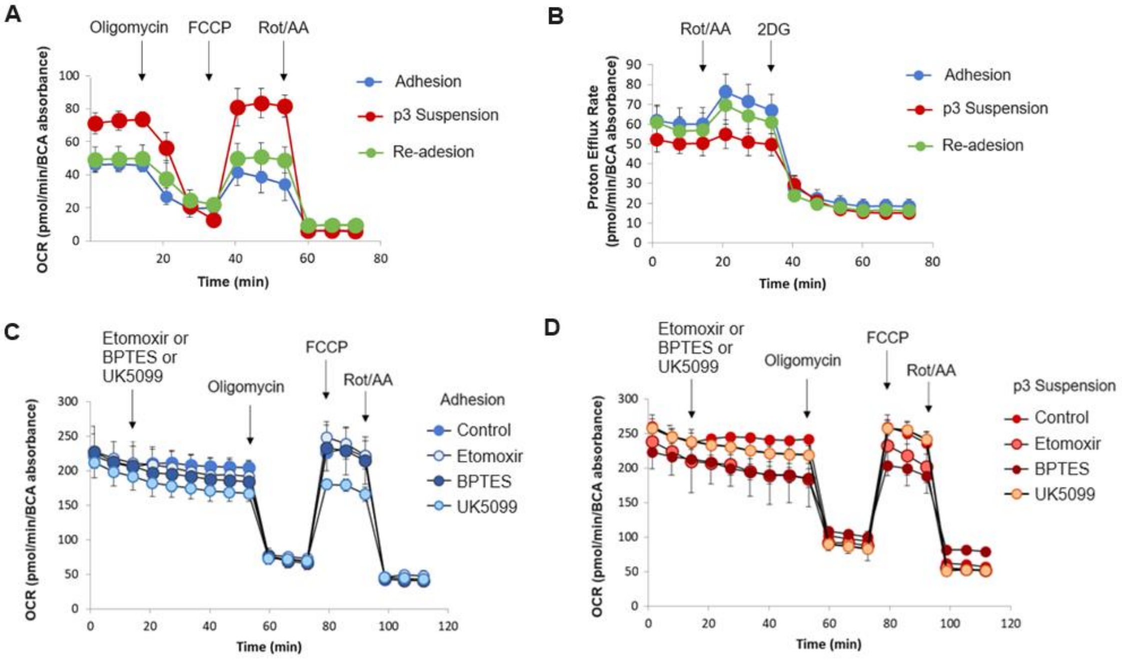

Figure S8- Representative time-course traces of OCR and Proton Efflux Rate of A375M6 melanoma cells, measured by the Seahorse XF Analyzer. OCR (A) and Proton Efflux Rate (B) of A375M6 grown in standard adherent conditions (Blu), p3 A375M6 cells subjected to 24 hours of rocking conditions (red) or p3 A375M6 cells after re-adhesion (green). Change in OCR of A375M6 grown in standard adherent conditions (C) and p3 A375M6 cells subjected to 24 hours of rocking conditions (D) after the injection of the indicated inhibitors: UK5099 inhibits glucose oxidation; etomoxir inhibits fatty acid oxidation; BPTES inhibits glutamine oxidations.

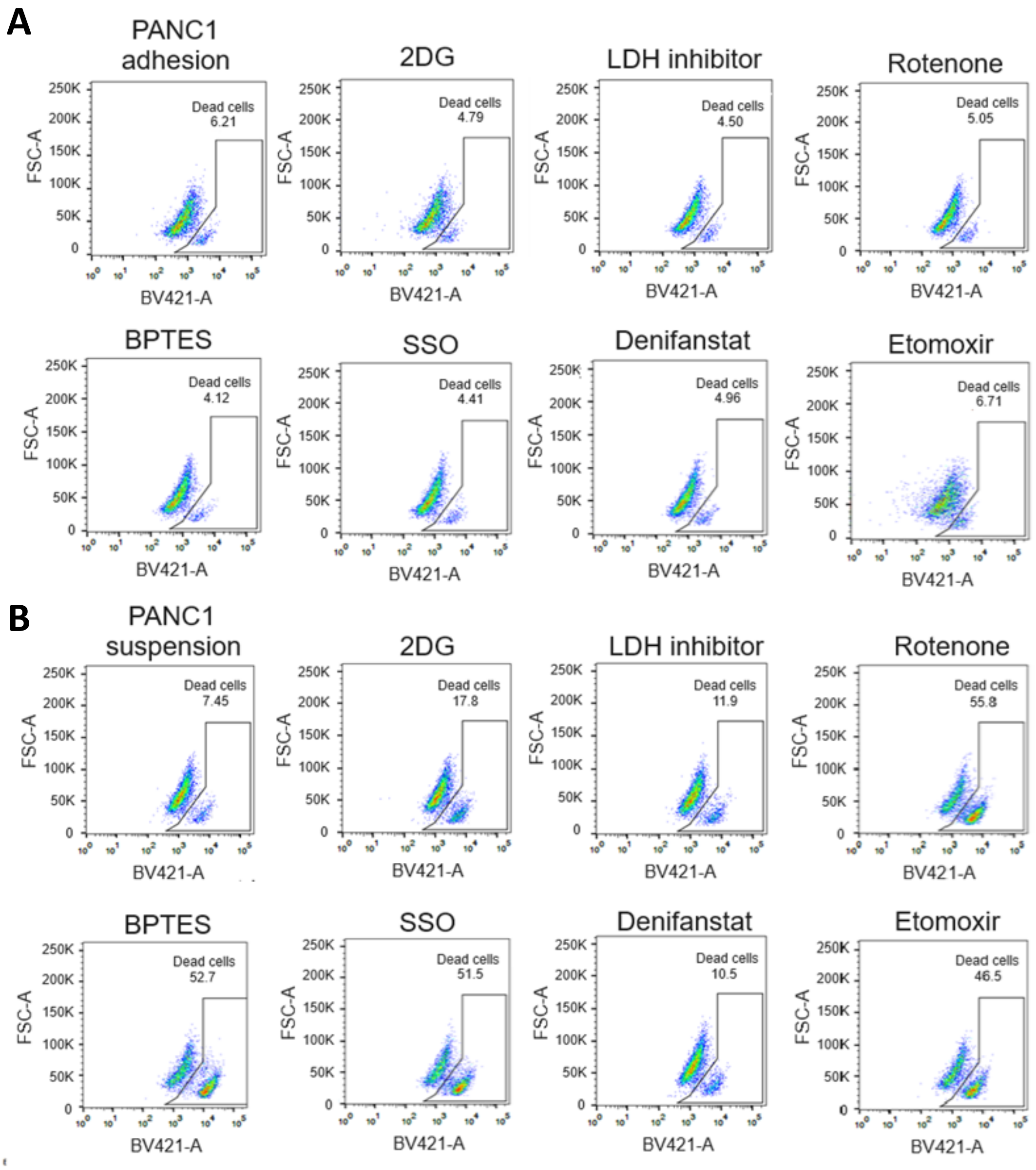

Figure S9- Representative plots of LIVE/DEAD Fixable Violet Dead Cell Staining of PANC1 pancreatic cancer cells under adherent (adhesion) (A) or rocking conditions (Suspension) (B), treated for 24 hours with 2DG, LDH-in-3, rotenone, BPTES, SSO, denifanstat or etomoxir.

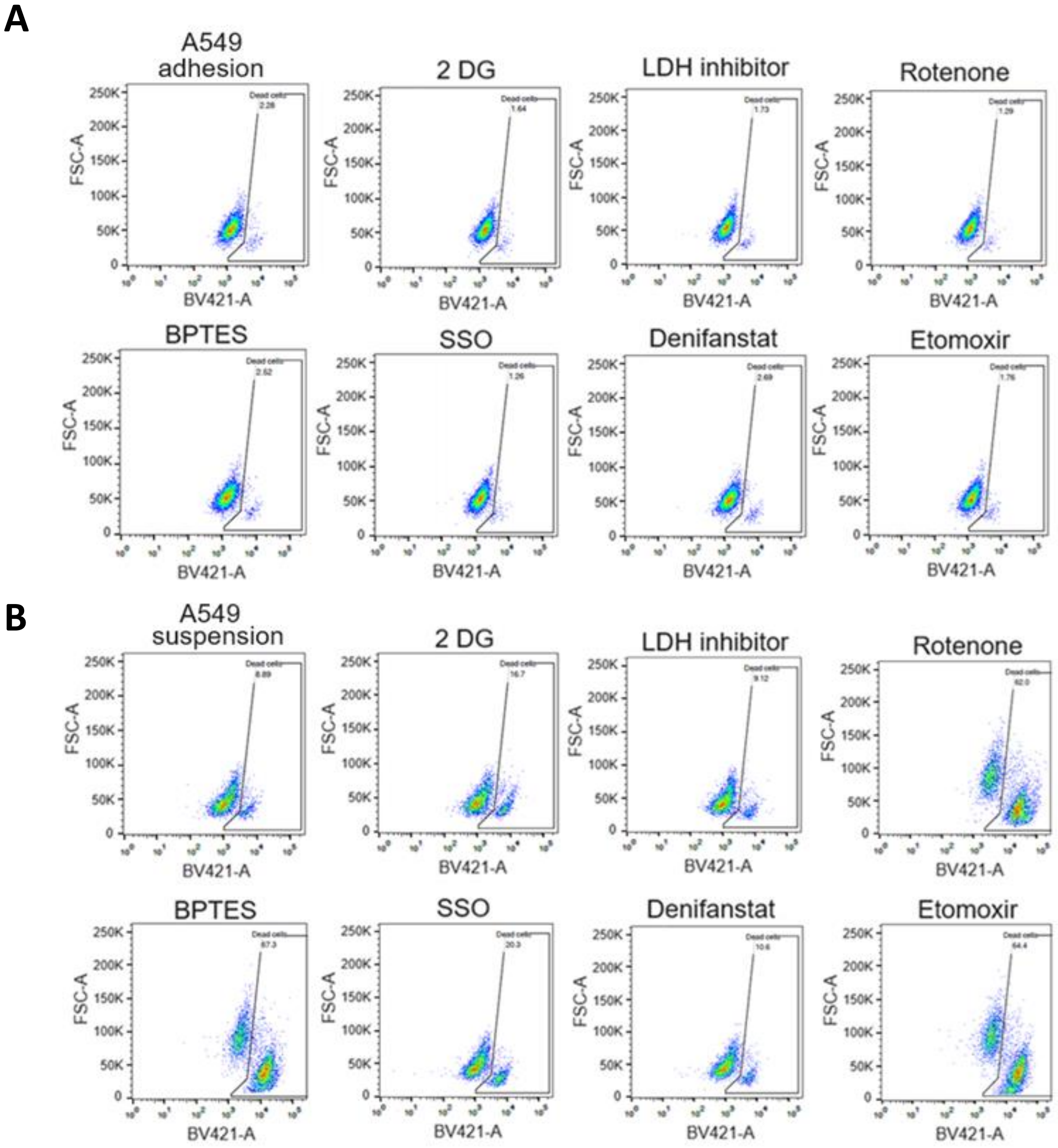

Figure S10- Representative plots of LIVE/DEAD Fixable Violet Dead Cell Staining of adenocarcinomic human alveolar basal epithelial A549 cells under adherent (adhesion) (A) or rocking conditions (Suspension) (B), treated for 24 hours with 2DG, LDH-in-3, rotenone, BPTES, SSO, denifanstat or etomoxir.
